# Peritumoral CD90+CD73+ cells possess immunosuppressive features in human non-small cell lung cancer

**DOI:** 10.1016/j.ebiom.2021.103664

**Published:** 2021-11-02

**Authors:** Limei Wang, Haitang Yang, Patrick Dorn, Sabina Berezowska, Fabian Blank, Carlos Wotzkow, Thomas M. Marti, Ren-Wang Peng, Nathalie Harrer, Wolfgang Sommergruber, Gregor J. Kocher, Ralph A. Schmid, Sean R.R. Hall

**Affiliations:** a Division of General Thoracic Surgery, Bern University Hospital, Bern, Switzerland; b Department of BioMedical Research, University of Bern, Murtenstrasse 50, Bern 3008, Switzerland; c Institute of Pathology, University of Bern, Switzerland; d DCR Live Imaging Core, University of Bern, Switzerland; e Boehringer Ingelheim, RCV GmbH & Co KG, Vienna, Austria

**Keywords:** Stroma, PD-L1, T cells, Immunosuppression, TGFβ1, Lung cancer

## Abstract

**Background:**

Although T cell abundance in solid tumours is associated with better outcomes, it also correlates with a stroma-mediated source of immune suppression driven by TGFβ1 and poor overall survival. Whether this also is observed in non-small cell lung cancer (NSCLC) is unknown.

**Methods:**

We utilized molecular analysis of The Cancer Genome Atlas (TCGA) NSLCC cohort to correlate immune activation (IA) gene expression and extracellular matrix/stromal (ECM/stromal) gene expression with patient survival. In an independent cohort of NSCLC samples, we used flow cytometry to identify mesenchymal subsets and *ex vivo* functional studies to characterize their immune regulatory function.

**Findings:**

We observed a high enrichment in a core set of genes defining an IA gene expression signature in NSCLC across TCGA Pan-cancer cohort. High IA signature score correlates with enrichment of ECM/stromal gene signature across TCGA NSCLC datasets. Importantly, a higher ratio of ECM/stromal to IA gene signature score was associated with shorter overall survival. In tumours resected from a separate cohort of NSCLC patients, we identified CD90+CD73+ peritumoral cells that were enriched in the ECM/stromal gene signature, which was amplified by TGFβ1. IFNγ and TNFα-primed peritumoral CD90+CD73+ cells upregulate immune checkpoint molecules PD-L1 and IDO1 and secrete an array of cytokines/chemokines including TGFβ1. Finally, immune primed peritumoral CD90+CD73+ cells suppress T cell function, which was relieved following combined blockade of PD-L1 and TGFβ1 with IDO1 inhibition but not PD-L1 or anti-CD73 alone.

**Interpretation:**

Our findings suggest that targeting PD-L1 together with independent biological features of the stroma may enhance host antitumor immunity in NSCLC.

**Funding:**

LW and HY are supported by a 4-year China Scholarship Council award. This work was funded, in part, by a grant from the Cancer League of Bern, Switzerland to SRRH. Laser scanning microscopy imaging was funded by the R'Equip grant from the Swiss National Science Foundation Nr. 316030_145003.

**Research in context**

**Evidence before this study**

Blockade of immune-checkpoint programmed death 1 (PD-1) or its cognate ligand PD-1 ligand (PD-L1) using antibodies has shown durable antitumor responses only in a minority of patients with advanced non-small cell lung cancer (NSCLC). Why the majority of patients fail to respond is not clearly understood. Emerging evidence in other solid tumor types such as melanoma and urothelial cancer show that T cell abundance correlates with a stroma-mediated source of immune resistance originating in transforming growth factor beta1 (TGFβ1)-responsive cancer-associated fibroblasts (CAFs). Importantly, this signature is linked with poor overall survival and resistance to immune checkpoint inhibitors. Whether CAFs or other mesenchymal cell types contribute to a tumour cell-extrinsic resistance signature in NSCLC is not known.

**Added value of this study**

We curated a core set of genes defining an immune activated (IA) signature and explored the relationship between this IA signature and ECM/stromal signature expressed by mesenchymal cells within the tumour microenvironment of various solid tumours using molecular analysis of The Cancer Gene Atlas (TCGA) NSCLC dataset. We observed a high enrichment of the IA gene signature in NSCLC and across TCGA Pan-cancer cohort. These immunologically active tumours showed a positive correlation with ECM/stromal gene expression. However, a higher ECM/stromal to IA signature ratio was associated with worse patient survival. To explore the potential source of the ECM/stromal gene signature in NSCLC, we used multiparametric flow cytometry and were able to identify a subset of non-hematopoietic, non-endothelial cells that were marked by CD90 and CD73 within tumour digests obtained from NSCLC patients undergoing resection for curative intent. These cells overexpress PD-L1, were peritumoral and enriched in the ECM/stromal gene signature, which was amplified by TGFβ1. Peritumoral CD90+CD73+ possess immunosuppressive features, unleashed following exposure to pro-inflammatory cytokines TNFα and IFNγ, potently suppressing T cell function using both cell contact and secreted soluble factors. Combined blockade of PD-L1, TGFβ1 and indoleamine 2,3-dioxygenase 1 (IDO1) rather than PD-L1 or CD73 blockade alone was able to restore the function of T cells.

**Implications of all the available evidence**

Our findings in this study raise the possibility that peritumoral CD90+CD73+ mesenchymal cells may perform an immune sentinel function in highly inflamed tumours and contribute to tumour cell-extrinsic negative regulation of host immunity. Whether this distinct cell subset gives rise to CAFs is not known, as CD90+CD73+ peritumoral cells were also a major source of inflammatory-driven IL-6, which was previously shown in NSCLC to be secreted from CAFs. Second, future preclinical studies using intact organisms will be required to determine whether there is a causal link between targeting the immune suppressive features of the stroma and enhanced antitumor immunity and response to immune checkpoint blockade in NSCLC.

Alt-text: Unlabelled box

## Introduction

The introduction of novel therapies aimed at boosting the host immune response against the tumour by blockade of the immune checkpoint molecule programmed cell death receptor 1 (PD-1) or its cognate ligand programmed cell death ligand 1 (PD-L1) has shown durable clinical responses in first and second line setting in a subset of patients with advanced non-small cell lung cancer (NSCLC).^1^, ^2^, ^3^, ^4^, ^5^ Despite this, the majority of advanced NSCLC patients derive no clinical benefit from immune checkpoint blockade (ICB) targeting the PD-1/PD-L1 axis.^6^ Interferon-gamma (IFN-γ) released by tumour-reactive infiltrating lymphocytes (TILs) represents a major driver of tumour cell-extrinsic PD-L1 expression and immune escape. Targets of IFN-γ signalling in the tumour microenvironment (TME) release additional molecules such as indoleamine 2,3-dioxygenase 1 (IDO1)^7^ that may serve as an additional brake on chronic T cell activation to restore immune host balance and prevent tissue pathology.^8^ In the setting of malignancy, IFN-γ-driven tumour cell-extrinsic mechanisms may contribute, in part, to immune resistance.^9^

NSCLC is divided into adenocarcinoma (LUAD) and squamous cell carcinoma (LUSC) based on their histological features.^10^ However, both LUAD and LUSC form heterogeneous, complex organ-like systems, whereby tumour cells interact with and adapt to a dynamic TME composed of an extracellular matrix (ECM) containing a heterogeneous mix of immune cells, lymphatics, blood vessels with their perivascular supporting cells and fibroblasts.^11^ The TME is a salient feature of most carcinoma-derived solid tumours, including NSCLC.^12^ Cancer-associated fibroblasts (CAFs) that stain positive for fibroblast activation protein-α (FAP) represent the predominant non-hematopoietic mesenchymal cell type of the reactive stroma found in carcinomas.^13^ In human lung tumours, the stromal ECM was shown to regulate both the localization and migration of tumour-reactive T cells thereby influencing antitumor immunity.^14^ A comprehensive molecular analysis of melanoma tumours in humans demonstrated a strong positive correlation between the expression of a distinct set of CAF-related genes and T cell infiltration.^15^ Although tumours enriched with T cells are associated with better overall survival,^16^ a stroma-mediated source of immune resistance originating in transforming growth factor beta1 (TGFβ1)-responsive CAFs is linked with poor overall survival and resistance to ICB targeting PD-1/PD-L1.^17^, ^18^, ^19^ However, the origin of CAFs in solid tumors is unclear. Lineage tracing in murines showed that pericytes transition to CAFs.^20^^,^^21^ Whether CAFs or other mesenchymal cell types present in the TME in NSCLC contribute to this tumor cell-extrinsic resistance signature in NSCLC has not been investigated.

Here, we curated a core set of genes defining an immune activation (IA) CD8+ T effector cell signature^22^, ^23^, ^24^ to explore the relationship with a curated ECM/stromal signature expressed by mesenchymal cells, including but not limited to CAFs, within the TME across various solid tumours^15^^,^^17^^,^^19^ using molecular analysis of The Cancer Gene Atlas (TCGA). We use these signatures to demonstrate that enrichment of the IA gene signature in NSCLC and across TCGA Pan-cancer cohort showed a positive correlation with ECM/stromal gene expression. In particular, a higher ECM/stromal to IA ratio is associated with worse patient survival in NSCLC. We use freshly resected tumor material from NSCLC patients to show that mesenchymal cells within the TME marked by CD90 and CD73 are enriched in the ECM/stromal gene signature amplified by TGFβ1 and are potently immunosuppressive. Finally, we demonstrate that combined blockade of signalling ligands PD-L1 and TGFβ1 together with inhibition of indoleamine 2,3-dioxygenase 1 (IDO1) was able to reverse, in part, the suppressive effects of peritumoral CD90+CD73+ cells on tumor-infiltrating lymphocytes (TILs).

## Methods

### Study approval and acquisition of tissue samples

The study was approved by Ethics Commission of the Canton of Bern (KEK-BE:042/2015). All patients gave informed written consent for use of surgical material for research purposes. Lung tumour samples were obtained from patients operated on for NSCLC with a curative intent at Bern University Hospital, Division of General Thoracic Surgery, September 2013 to January 2019. Unfixed surgical specimens were sent to the Institute of Pathology, University of Bern, where a pathologist dissected the tumor and matched non-tumorous lung tissue for further analysis. Cases included in the study were diagnosed as either LUAD (n = 64) or LUSC (n = 59). Twenty-nine patients received neoadjuvant treatment. Further clinic-pathological characteristics are provided in Table S1

### The Cancer Genome Atlas (TCGA) database and establishment of ECM and IIA gene signatures

To generate an RNA-based metric to score ECM/stroma, we defined a gene set that contributes to the ECM, epithelial-to-mesenchymal transition (EMT) or stroma where there would be overlap in the expression found in the two main mesenchymal cell subsets in solid tumours: cancer-associated fibroblasts (CAFs) and pericytes. We chose hallmark CAF/pericyte genes VCAN, FAP, COL1A1, POSTN, THY1, FBLN1, and TGFβ1 mined from three sources.^15^^,^^17^^,^^19^ We also choose genes IL6, CSPG4, PDPN, HGF, SERPINE1 specific to lung perivascular-like cells.^25^^,^^26^ The defined gene set for the immune activation (IA) signature was chosen using hallmark genes with cytolytic activity expressed by CD8 TILs (GZMA, GZMB, GZMK, PRF1, IFNG, GNLY and IL2) [22,24] and global activation of CD8 TILs based on single-cell RNA sequencing data in lung cancer patients.^23^ Transcriptomic data were obtained from The Cancer Genome Atlas (TCGA) (https://portal.gdc.cancer.gov/projects/TCGA). After normalization (Limma package in the R) and log2 transformation, transcriptomic data were subjected to further analysis. Gene signature sore calculation: after scaling the genes expression value by Apply function in R, a sum of gene expression of the selected genes within the gene signature was then summarised as a single score for each sample. The gene expression and corresponding survival data were extracted for correlation and prognostic analysis using the corresponding packages in R (´corrplot´ and ´Hmisc´ packages for correlation analysis; 'maxstat', 'survival' and 'survminer' packages for prognostic analysis).

### Flow cytometric profiling and prospective cell isolation

Generation of single-cell suspensions from tumour and matched non-tumorous lung tissue for flow cytometric profiling and prospective cell isolation using fluorescence-activated cell sorting (FACS) have been previously described.^27^ Briefly, single cells were stained in buffer with Fc block (eBioscience) containing a panel of fluorescently conjugated human monoclonal antibodies directed at the following epitopes: CD45, CD14, CD31, CD235a into one channel, CD73, CD90, PD-L1, CD47 and EpCAM (see Table S2). To exclude cells from the analysis that stain for lineage markers not expressed by mesenchymal or epithelial cells, we created a dump gate pooling CD45, CD14, CD31, CD235a into one channel (see Fig. S4 for full gating strategy). In a second smaller cohort, single cells were stained with a second antibody panel: CD45, CD14, CD235a, CD31, CD73, CD90, CD39, PD-L1, PDGFRα and EpCAM (see Table S2). To exclude cells from the analysis that stain for lineage markers not expressed by mesenchymal, endothelial or epithelial cells, we created a dump gate pooling CD45, CD14, CD235a into one channel. Flow cytometric profiling of the mesenchymal compartment of single-cell digests of tumour and matched uninvolved lung tissue was performed using a BD FACS LSRII (BD Biosciences). For analysis, a minimum 5 × 10^5^ live events were collected and analyzed using FlowJo software ver10.7.1. PD-L1 and CD47 expression were measured as geometric mean fluorescence intensity (gMFI) in FlowJo. To prospectively isolate mesenchymal cells from the tumour and matched uninvolved tissue, single cells were stained as described above and sorted directly into collection buffer containing 20% FBS using a BD FACS Aria III or BD FACS Aria. Following this, cells were expanded in α-MEM (Sigma) supplemented with 1% FBS (Invitrogen), 10 ng/ml of recombinant human bFGF (Gibco, Invitrogen), 20 ng/ml of recombinant EGF (Gibco, Invitrogen) and 1.25 mg of human insulin solution (Sigma) and 1X antimycotic/antibiotic (Gibco, Invitrogen). Culture expanded cells were used for all downstream experiments (See Supplemental Materials and Methods for details).

### Statistical analysis

Data are expressed as mean ± SD. Comparisons between two groups were carried out using the parametric student's two-tailed paired or unpaired t-test for normally distributed data. If data were not distributed normally, a nonparametric Wilcoxon signed-rank test was used between the two groups. One-way analysis of variance (ANOVA) followed by post hoc Tukey’ range test was used for analysis of more than two groups. The numbers of samples (biological replicates) per group (n), or the numbers of experiments (technical replicates) are specified in the figure legends. Data were analyzed using GraphPad Prism 8 software. For survival analysis, patients were grouped by gene expression, where ‘high’ and ‘low’ expression groups were stratified by the optimal cutoff value. Then, Kapler-Meier analysis of a TCGA cohort of patients with LUSC and LUAD was performed. Stratification of patients into high_extracellular matrix/stromal (ECM/stromal) (in red) and low_ECM/stromal (in black) or high_IA and low_IA is based on the optimal cutoff value of ECM/stromal gene signature score transcripts across all patients by using the surv_cutpoint function in R 'maxstat' package. Overall survival curves and cumulative hazard rates were analyzed and plotted by using R 'survival' and 'survminer' packages. The p-value is calculated using the log-rank test. All other statistical analyses were performed in the R Statistical Computing environment v3.3.1 (http://www.r-project.org). Statistical significance is accepted at p < 0.05.

#### Role of funding source

The Funders had no role in the study design, data collection, data analyses, interpretation, or writing of report, and the decision of paper submission.

## Results

### ECM/stromal gene signature is correlated with PD-L1 expression and CD8 T effector cell phenotypic gene signature in NSCLC

**Figure 1:**
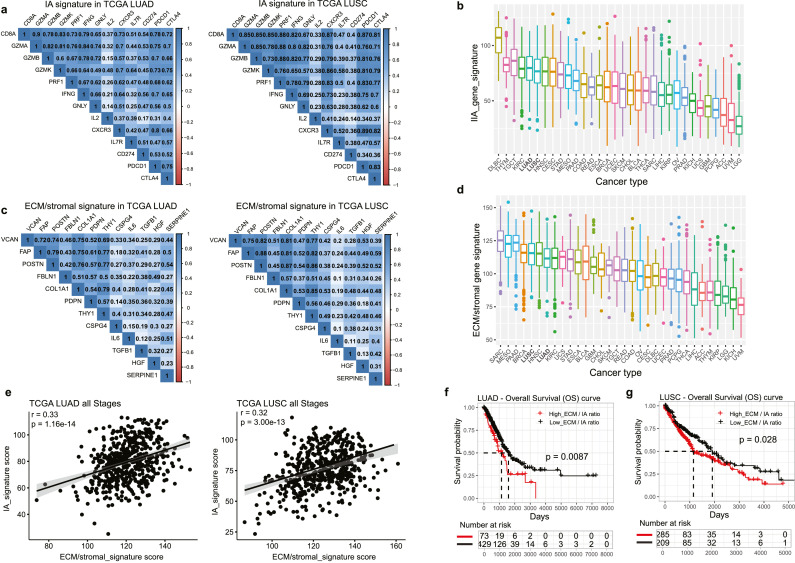
**ECM/stromal genes and CD8 T cell activation genes are associated with NSCLC tumorigenesis, as well as prognosi**s. (a) Correlation analysis of the individual genes in immune infiltration/activation (IA) gene set across TCGA LUAD and LUSC cohorts. Transcriptomic data of lung cancer patients were obtained from The Cancer Genome Atlas (TCGA) (https://portal.gdc.cancer.gov/projects/TCGA). The numbers in the correlogram indicate the correlation coefficient (Spearman). Significant positive (in blue) and negative (in red) correlations are shown, with color intensity proportional to the correlation coefficient. Non-significant correlation is left as a blank background. P-value < 0.05 is considered significant. (b) Boxplots showing IA gene signature scores across various cancer types in TCGA. (c) Correlation analysis of the individual genes in extracellular matrix (ECM)/stromal gene set across TCGA LUAD and LUSC cohorts. (d) Boxplots showing ECM/stromal signature scores across various cancer types in TCGA. (e) Plots showing the correlation between ECM/stromal gene signature with IA signature in TCGA LUAD and LUSC cohorts. (f,g) Unadjusted Kaplan-Meier curves showing overall survival (OS) by the ratio of ECM/stromal to IA gene expression in LUAD (f) and LUSC (g).

Expression of PD-L1 represents an adaptive response to proinflammatory molecules IFNγ and TNFα released from tumour-reactive effector T cells^28^ and represents a predictive biomarker for ICB in NSCLC; although this remains controversial.^29^ As recently shown, IFNγ-driven gene signatures may represent a better prognostic indicator for overall patient survival.^22^^,^^30^ To investigate this in NSCLC, we generated a 13-gene CD8+ T effector cell phenotypic immune activation (IA) signature consisting of a core set of cytotoxic effector molecules (IFN-γ, GMZA, GZMB, GZMK, PRF1, GNLY), markers of activation (IL-2, PDCD1 (PD-1), CD274 (PD-L1), and CTLA4) together with infiltration (CXCR3) and homeostatic regulation (IL7R or CD127). The genes within the defined IA signature show a mutually significant positive correlation in TCGA LUAD and LUSC datasets (Fig. 1a), indicating a similar immunoregulatory pattern among genes in the set. We also observed a high enrichment in the IA signature score in LUAD and LUSC samples across TCGA Pan-cancer cohort, suggesting an enriched CD8+ T effector cell phenotype (Fig. 1b). Previous studies have shown a positive correlation between T cell abundance and epithelial-to-mesenchyme (EMT) and stromal-specific gene expression profile originating in non-tumour stroma cells rather than cancer cells in melanoma and urothelial cancer.^15^^,^^19^ To explore this correlation in NSCLC, we defined a distinct ECM/stromal transcriptional signature consisting of 12 genes known to be expressed by mesenchymal cells within the TME. These 12 genes were part of a larger TGFβ1-driven gene signature in CAFs that highly correlated with T cell infiltration.^15^^,^^17^^,^^19^ Within this core signature, we show a mutually significant positive correlation across LUAD and LUSC cohorts (Fig. 1c). In addition, ECM/stromal gene signature scores were enriched in LUAD and LUSC samples across TCGA Pan-cancer cohort (Fig. 1d). Next, we validated a positive correlation between ECM/stromal gene signature score and IA signature score across TCGA LUAD and LUSC dataset (Fig. 1e), which is independent of tumour stages (Fig. S1a,b). High ECM/stromal gene expression alone was not associated with worse patient survival in LUAD, which was in contrast with LUSC where a high ECM/stromal gene score was associated with worse survival (Fig. S1c,d). For both LUAD and LUSC, a high IA gene expression signature score alone was associated with longer patient survival (Fig. S1e,f). When we performed survival analysis using a ratio of ECM/stromal and IA gene signature scores, we observed that a higher ratio of ECM/stromal to IA gene signature score was highly associated with shorter survival in TCGA LUAD and LUSC cohorts (Fig. 1f,g). Collectively, these analyses suggest that the ECM/stromal-defined TME might represent a critical tumour cell-extrinsic regulator of tumour immunity in NSCLC.

### Peritumoral CD90 and CD73 mesenchymal cells are associated with the ECM/stromal gene-set and immunologically active tumors

**Figure 2:**
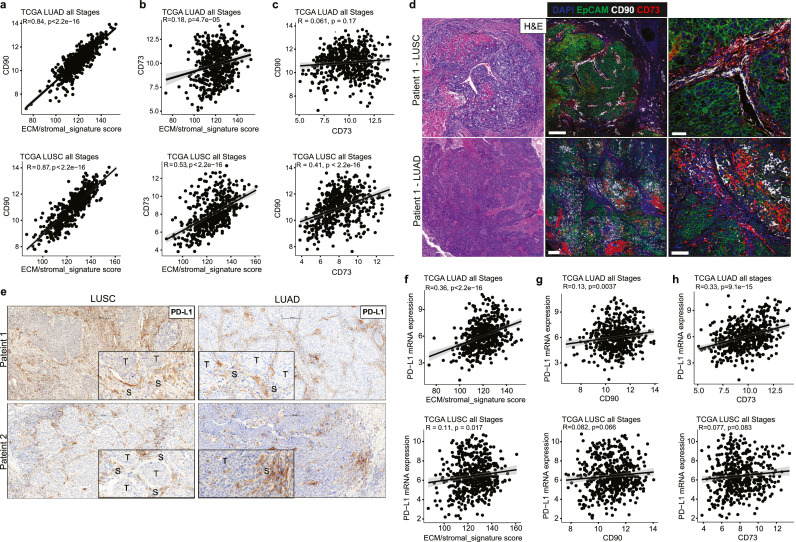
**Peritumoral cells marked by CD90 and CD73 in NSCLC**. (a,b) Plots showing the correlation between CD90 (a) and CD73 (b) mRNA expression with the ECM/stromal gene signature score in TCGA LUAD and LUSC cohorts. (c) Plots showing the correlation between CD90 and CD73 in TCGA LUAD and LUSC cohorts. (d) Representative H&E and corresponding confocal images showing the location of CD90 (white), CD73 (red) cells in relationship to EpCAM (green) positive tumour islands. Nuclei are pseudocoloured blue. Scale bar 50 µm. (e) Representative images of PD-L1 expression in NSCLC patient specimens. Scale bar 200 µm. (f) Plots showing the correlation between PD-L1 mRNA with the ECM/stromal gene signature score in TCGA LUAD and LUSC cohorts. (g,h) Plots showing the correlation between PD-L1 with CD90 (g) and CD73 (h) mRNA expression in TCGA LUAD and LUSC cohorts.

Seminal observations from the PanCan Atlas consortium of over 33 cancer types, including LUAD and LUSC, suggest that both the spatial distribution and immune cell composition are determined largely by the cellular makeup of the TME.^31^^,^^32^ We have previously identified a subset of mesenchymal cells co-expressing thymocyte differentiation antigen 1 (*THY1*, encoding CD90) and the ecto-5′-nucleotidase (*NT5E*, encoding CD73) with an abnormal perivascular-like function that also expressed PD-L1 in human NSCLC.^27^ We validated a high correlation between CD90 mRNA level and the ECM/stromal signature score across TCGA LUAD and LUSC cohorts (Fig. 2a). CD73 mRNA expression also positively correlates with the ECM/stromal signature score but to a weaker extent (Fig. 2b). A similar trend was also observed between CD90 and CD73 expression with IA gene signature score (Fig. S2a,b). Finally, no correlation between CD90 and CD73 in LUAD (Spearman's ρ, p = 0.06) was observed, in contrast to LUSC (Spearman's ρ, p = 0.41, Fig. 2c). This may indicate a spatial distribution between CD90 and CD73 marked stromal cell populations. Based on univariate analysis, high gene expression of CD90 was associated with worse overall survival in LUSC (p = 0.011) but not in LUAD (p = 0.11) (Fig. S2c), whereas high expression of CD73 was associated with worse patient survival in both LUAD (p = 0.0051) and LUSC (p = 6e-04) (Fig. S2d).

Next, we examined the spatial distribution of CD90 and CD73 in relationship with tumour epithelium. Confocal imaging showed CD90 and CD73 positive cells marking different mesenchymal cell populations surrounding epithelial cell adhesion molecule (EpCAM) positive tumour islands (Figs. 2d and S2e). Although primarily described as being expressed by tumour or immune cells, PD-L1 expression can be observed in the tumour stroma (Fig. 2e). Overall, there was a strong correlation in PD-L1 at the protein and mRNA level in LUAD and LUSC and across the Pan-cancer TGCA cohort (Fig. S3). There was a positive correlation between PD-L1 expression and the ECM/stromal gene signature in LUAD but not LUSC (Fig. 2f). We also observed a weaker correlation between CD90 and CD73 with PD-L1 in TCGA LUAD, whereas no correlation was observed in LUSC cohorts (Fig. 2g,h). Collectively, these data suggest that CD90 and CD73 cells contained within the nontumor stroma may represent an important contributor to local immune regulation in NSCLC.

### Expression of immune checkpoint molecules in mesenchymal cell subsets in NSCLC

To identify the cellular source of ECM/stromal gene signature, we applied our previously developed multiparametric flow cytometric profiling strategy in resected tumours from a larger cohort of human NSCLC patients for the presence of mesenchymal cells based on the expression of CD90 and CD73 and their coexpression of PD-L1 and the innate checkpoint molecule CD47 (Fig. S4a,b).^27^ In tumor (T) and matched uninvolved nontumor (N) digests, the predominant mesenchymal cell subset was CD90-CD73- in both LUAD (N, 76%±16% vs T, 69.8%±22%, p = 0.064) and LUSC (N, 73%±20% vs T, 67%±26%, p = 0.1) (Fig. S4c,d), which expressed low levels of PD-L1 in tumor (Fig. S4c,d). In contrast, PD-L1 overexpression was observed in CD90+CD73+ and CD90+CD73- tumor mesenchymal subsets in both LUAD (N, 506±570 vs T, 891±1263, p < 0.0001; and N, 309±386 vs T, 580±1088, p < 0.0001, respectively) and LUSC (N, 684±632 vs T, 1054±1237, p = 0.0013; and N, 391±448 vs T, 596±979, p = 0.0027, respectively) (Fig. S4e,f). There was a high negative correlation between % of CD90-CD73- and CD90+CD73+ cells in both LUAD and LUSC (Fig. S4g–i).

**Figure 3:**
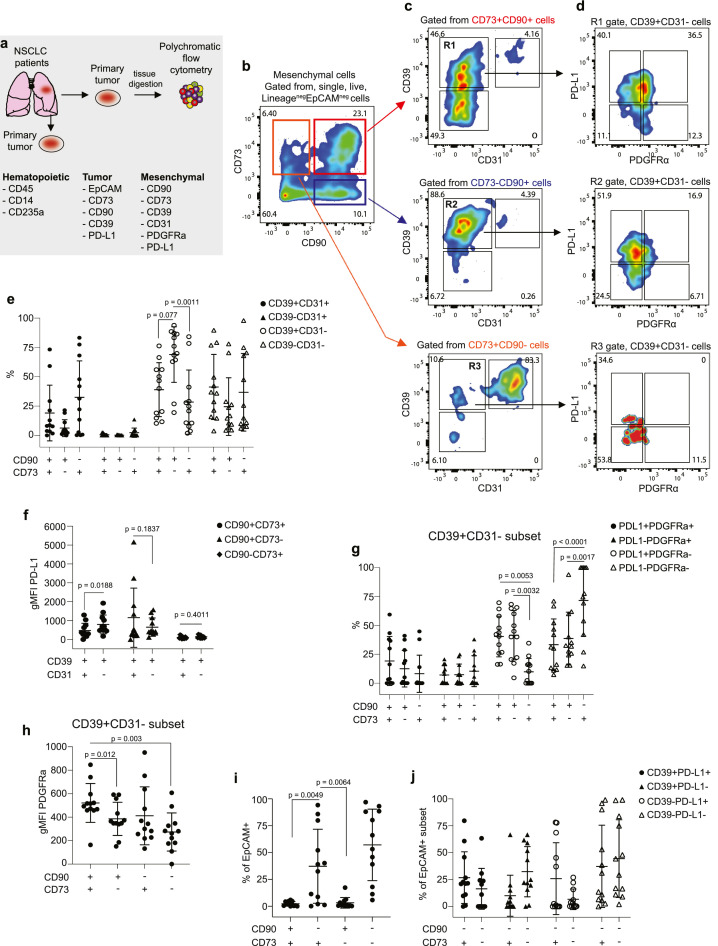
**Heterogeneity in the expression of CD39, PD-L1 and PDGFR**α **in mesenchymal cell subsets in NSCLC.** (a) Schematic showing antibody panel used for characterization of mesenchymal cell subsets using multiparametric flow cytometry. (b) Representative bivariate plot showing mesenchymal fraction subgated for CD90 and CD73. (c) Colour-coded mesenchymal fractions were further subgated onto bivariate plots for CD39 and CD31. (d) Selected populations in R1 and R2 gates were further subgated onto bivariate plots for PD-L1 and PDGFRα. (e) Frequency of CD90 and CD73 mesenchymal subsets co-expressing CD39 and CD31 in NSCLC specimens (n = 12). (f) Scatter plots showing geometric mean fluorescence intensity (gMFI) for PD-L1 on gated mesenchymal cell subsets. (g) Frequency of mesenchymal subsets based on CD39+CD31- (top panel) co-expressing PD-L1 and PDGFRα expression. (h) Scatter plots showing gMFI for PDGFRα on gated mesenchymal cell subsets co-expressing CD39. (i) Frequency of EpCAM+ tumour cells co-expressing CD73 and CD90. (j) Frequency of EpCAM+ tumour cell subsets co-expressing CD39 and PD-L1. All data determined by flow cytometry. Data presented as mean ± SD. Significant differences in e, g, and i calculated using two-way ANOVA followed by following by post hoc Tukey's range test. Significant differences in f calculated using a two-tailed, student's paired t-test.

CD73 together with another cell surface ectonucleotidase CD39 convert extracellular ATP to immunosuppressive adenosine contributing to an immunosuppressive TME.^33^ Together with PD-L1, CD73 and CD39 are broadly expressed on the tumor epithelium, stroma and vasculature. To address this further, we used flow cytometry to investigate the expression of these checkpoint molecules in tumor epithelium (EpCAM)_compared with mesenchymal cell susbets and vasculature (CD31) in a smaller cohort of patients (n = 12) (Fig. 3a). Using a sequential gating strategy (Fig. 3b,-d), we showed that 39±23% of CD90+CD73+ cells co-expressed CD39 but lacked CD31, whereas 0.6±1% of CD90+CD73+ co-expressed CD31 while lacking CD39 (Fig. 3e). We also found that 19.1±23.7% of CD90+CD73+ co-expressed both CD39 and CD31. In comparison, 69±24% of the CD90+CD73- mesenchymal subset was found to co-express CD39 while lacking CD31 whereas only 6.2±7% of this subset co-expressed both CD39 and CD31. Whereas 28±27% of the CD90-CD73+ mesenchymal cell subset co-expressed CD39 only compared with 32.5±31% coexpressing both CD39 and CD31. We further observed an upregulation in PD-L1 expression in CD90+CD73+CD39+CD31- compared with CD90+CD73+CD39+CD31+ cells (Fig. 3f, left panel), which was not observed in the CD90+CD73- (Fig. 3f, centre panel) or CD90-CD73+ mesenchymal cell subsets (Fig. 3f, right panel). Moreover, there was a small percentage of mesenchymal cell susbets that co-expressed PDGFRα mainly observed in the CD90+CD73+CD39+ and CD90+CD73-CD39+ cell subsets (Fig. 3g, h). EpCAM+ tumour cells that co-express CD73 (Fig. S3h) were also found to express CD39 and PD-L1 (Fig. 3i,j). Our data confirm the significant phenotypic heterogeneity in the mesenchymal compartment that has been reported in NSCLC.^34^

### ECM/stromal signature is enriched in peritumoral CD90+CD73+ mesenchymal cells and amplified by TGFβ1

**Figure 4:**
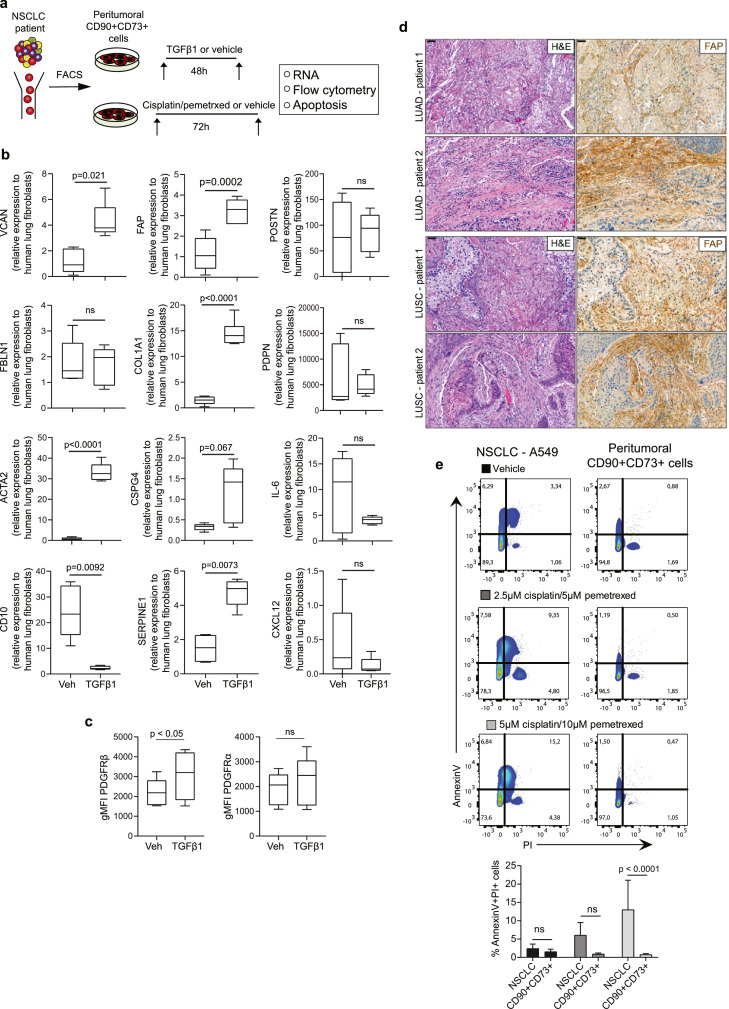
**Peritumoral CD90+CD73+ cells enriched in ECM/stromal gene expression signature are resistant to chemotherapy**. (a) Schematic showing methodology used to characterize peritumoral CD90+CD73+ cells. (b) Box plots showing the change in mRNA expression in a distinct ECM/stromal gene set in response to TGFβ1. mRNA level in adult lung human fibroblasts is set at one. n = 10, biological replicates in total. LUAD, n = 5; LUSC, n = 5. (c) Box plots showing the change in PDGFRα (left) and PDGFRβ (right) geometric mean fluorescent intensity (gMFI) in response to TGFβ1. n = 10, biological replicates in total. n = 5, LUAD; n = 5, LUAD. (d) Representative H&E (left) and FAP expression (right) in serial sections in LUAD and LUSC patients. Scale bar 200µm. (e) Bar graph showing the quantification of double positive Annexin V/PI stained cells. NSCLC cell lines, n = 4, (LUAD: A549, H1299 and LUSC: H520, H1703); peritumoral CD90+CD73+ cells, n = 8, biological replicates (LUAD, n =4; LUSC, n = 4). Data presented as mean ± SD. Significant differences in c using a two-tailed, student's paired t-test. Significant differences in b, e calculated using one way ANOVA following by post hoc Tukey's range test. ns, not significant.

Next, we prospectively isolated the CD90+CD73+ mesenchymal cell subset from both LUAD and LUSC tumour digests and exposed them to the pleiotropic cytokine TGFβ1 (Fig. 4a). At the mRNA level, genes comprising the ECM/stromal gene signature were enriched in peritumoral CD90+CD73+ cells and amplified by TGFβ1 (Fig. 4b). At the protein level, TGFβ1 upregulated the expression of PDGFRβ (p < 0.05, paired student's t-test) (Fig. 4c). The same trend was found with PDGFRα; however, this did not reach significance. We have previously demonstrated that tumour-associated CD90+CD73+ also express FAP at the mRNA level.^27^ Here, TGFβ1 increased FAP expression in CD90+CD73+ peritumoral cells (Fig. 4b) and FAP+ cells were observed adjacent to and surrounding tumour epithelium (Fig. 4d). To deconvolute the complexity of the mesenchymal compartment, we examined publicly available single-cell RNA sequencing data to validate the mesenchymal marker expression from patients with NSCLC^34^ and pancreatic adenocarcinoma (PAAD)^35^ downloaded from the Tumor Immune Single-cell Hub^36^ to determine the clustering across all cellular compartments. Single-cell analysis reveals enrichment of CD90 (THY1), CD73 (NT5E), and FAP in a cluster of fibroblasts in both NSCLC and PAAD (Fig. S5a,b). In our cohort, 28 patients underwent neoadjuvant chemotherapy prior to surgical resection. Although neoadjuvant chemotherapy had no effect on the mesenchymal cell subset composition or PD-L1 expression compared to non-treated patients (data not shown), treatment of CD90+CD73+ mesenchymal subset with conventional chemotherapy using cisplatin/pemetrexed did not result in a significant increase in Annexin V/PI staining (% 0.8±0.3) compared with NSCLC cell lines (% 13.1±8) (Fig. 4e). Collectively, these data suggest that the ECM/stromal gene signature may arise from peritumoral CD90+CD73+ cells, which we previously demonstrated dysregulated perivascular-like function^27^ and shown to be the cell of origin of CAFs in well-defined murine models of cancer.^20^^,^^21^

### Peritumoral CD90+CD73+ cells possess an immunoregulatory phenotype

**Figure 5:**
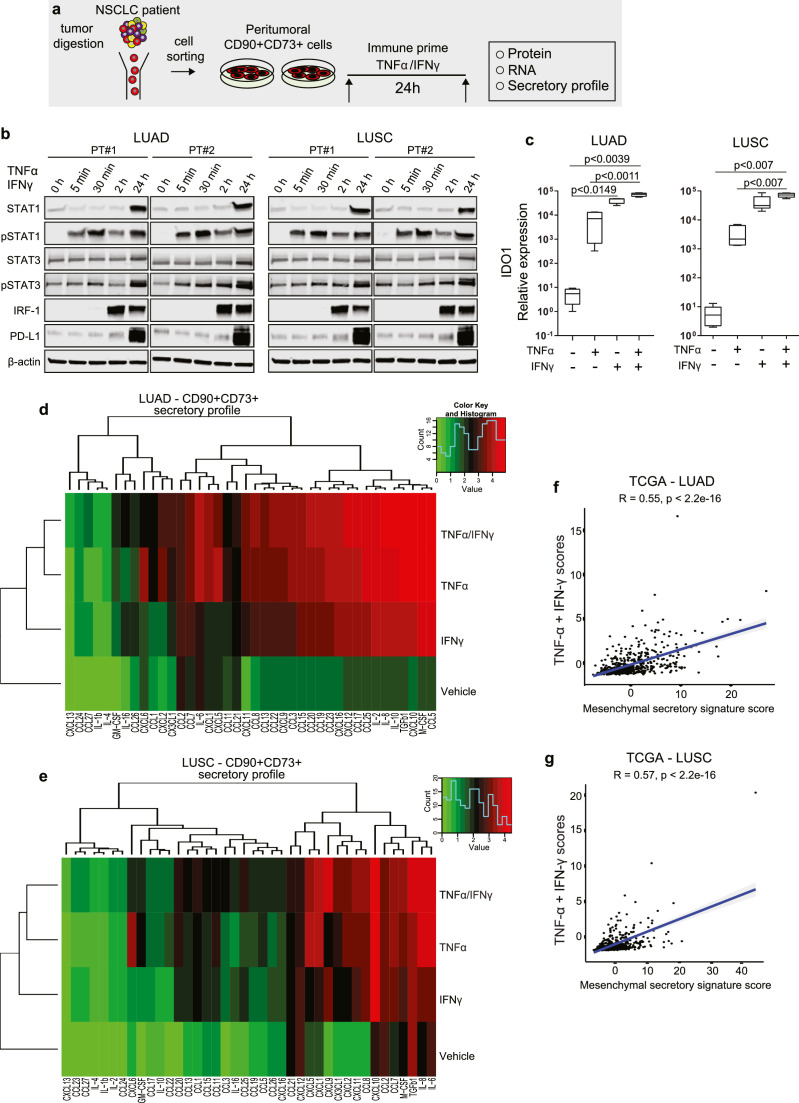
**Peritumoral CD90+CD73+ cells possess an immunoregulatory phenotype.** (a) Schematic outlining experimental setup to characterize the immunoregulatory phenotype of peritumoral CD90+CD73+ cells. (b) Western blots showing regulation of PD-L1 protein expression in peritumoral CD90+CD73+ cells via the JAK/STAT/IRF-1 pathway. Peritumoral CD90+CD73+ cells were immune primed with 50 ng/ml rhTNFα in combination with 50 ng/ml rhIFNγ simultaneously in serum-free media for indicated times. (c) Box plots for IDO1 mRNA expression in peritumoral CD90+CD73+ from both LUAD (n = 4) and LUSC (n =4) patients primed with vehicle, TNFα or INFγ or both cytokines simultaneously over 24 h. (d,e) Heatmap showing individual levels of proteins secreted by peritumoral CD90+CD73+ cells. Growth arrested peritumoral CD90+CD73+ mesenchymal cells from LUAD (n = 8) (d) and LUSC (n = 8) (e) tumour digests were treated with vehicle, TNFα or INFγ or both cytokines simultaneously over 24 h. Afterwards, factors secreted in the cell supernatants were detected. The colour represents the Tukey's ladder of powers transformation in R. (f,g) Scatter plots showing the correlation between TNFα and IFN-γ mRNA expression with mesenchymal cytokine score in TCGA LUAD (f) and LUSC (g) cohorts. Data presented in c as mean ± SD. Significant differences in c calculated using one ANOVA following by post hoc Tukey's range test.

It is reasonable to postulate that prior to their infiltration into tumour islands, tumour-reactive T cells would be in direct contact with peritumoral CD90+CD73+ cells in the stroma. Whether this distinct mesenchymal subset regulates local immune function is unknown. To investigate this, we exposed primary cultures of peritumoral CD90+CD73+ cells (LUSC, n = 8; and LUAD, n = 8, biological replicates) to TNFα and IFNγ, two pro-inflammatory cytokines that feature prominently in inflamed tumours (Fig. 5a). Here, PD-L1 was regulated at the protein level by the induction of proteins and phosphorylated proteins involved in the JAK-STAT signalling pathway including STAT1/pSTAT1, STAT3/pSTAT3 and increase expression of IRF-1 (Fig. 5b). IFNγ increases the expression of the immunomodulating heme enzyme indoleamine 2, 3 dioxygenase 1 (IDO1), which mediates depletion of tryptophan and suppression of T cell function.^37^ In both primary LUAD- and LUSC-derived peritumoral CD90+CD73+ cells, IDO1 was significantly elevated at the mRNA level following a single treatment with TNFα or IFNγ or in combination (Fig. 5c).

To identify factors secreted by CD90+CD73+ peritumoral cells, we performed an analysis of supernatants from immune primed peritumoral CD90+CD73+ cells, which revealed a wide range of secreted pro-inflammatory cytokines, C-C and C-X-C chemokines and growth factors that are critically involved in the recruitment, retention and functional modification of leukocytes (Fig. 5d,e). Secretion of 15 of 40 analyzed cytokines/chemokines was induced by TNFα only (> 5 fold) and included CCL1, CCL2, CCL5, CCL11, CCL13, CCL20, CCL22, CXCL1, CXCL2, CXCL5, M-CSF, GM-CSF, IL-6, IL-8 and IL-10. Whereas IFNγ enhanced the secretion (> 5 fold) of IL-16, IL-2, CCL25, CCL2 and CCL23. Combined exposure to TNFα and IFNγ resulted in synergistic increases in IL-16, CCL8, CX3CL1, CXCL9 and TGFβ1, whereas additive effects were found for CCL1, CCL7, CCL15, CCL17, CCL19, CCL24, CCL26, CXCL16, M-CSF and IL-2. TNFα or IFNγ alone increased the secretion of CXCL10, CXCL11 and CCL2; however, there was no change when TNFα and IFNγ were combined. There were notable differences in the types of cytokines and the amount secreted between LUAD and LUSC peritumoral CD90+CD73+ cells. Based on the top 10 secreted proteins, we generated a mesenchymal secretory signature and found that there was a positive correlation with the expression of TNFα and IFNγ in TCGA LUAD and LUSC dataset (Fig. 5f,g). Taken together, these data suggest that peritumoral CD90+CD73+ cells may be part of an important local negative feedback loop that performs immune sentinel function in an attempt to prevent extensive tissue damage while restoring homeostasis following injury-induced activation of T cells.

### Peritumoral CD90+CD73+ cells suppress T cells

**Figure 6:**
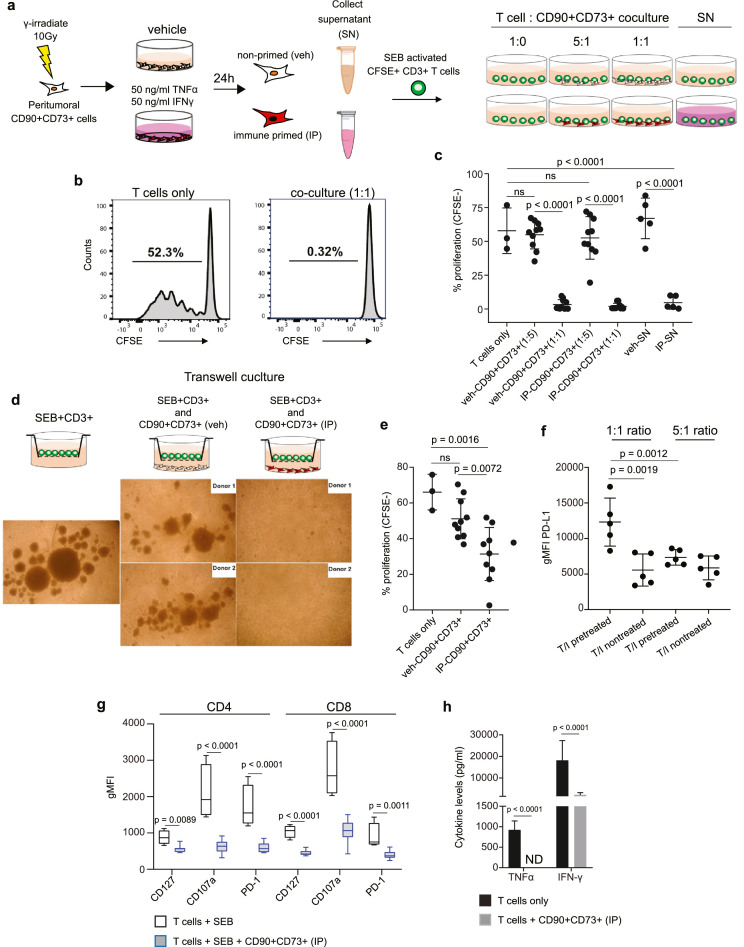
**Peritumoral CD90+CD73+ cells suppress T cells**. (a) Schematic of experimental set-up to examine immune suppressive function of peritumoral CD90+CD73+ cells. (b) Representative flow cytometric histogram showing proliferation of CFSE-labeled T cells isolated from the peripheral blood of a healthy donor 5 days following activation with staphylococcal enterotoxin B (SEB, 1 µg/mL) alone or in the presence of vehicle-treated or immune primed (IP) CD90+CD73+ cells at a 1:1 or 5:1 ratio (c) Scatter plots showing proliferation of CFSE-labeled T cells cocultured with vehicle or IP CD90+CD73+ cells, as well as their respective supernatants (SN). For coculture conditions n = 10 in total. LUAD, n = 5; LUSC, n = 5. For cell supernatant conditions n = 5 in total, n = 3 for LUSC and n = 2 for LUAD. (d) Schematic representation of transwell culture system with brightfield images of T cells 5 days following stimulation. (e) Scatter plots showing proliferation of CFSE-labeled T cells using a transwell system. n = 10 in total. n = 5 for LUSC and n = 5 for LUAD specimens. (f) Scatter plots showing the change in PD-L1 geometric mean fluorescence intensity (gMFI) in peritumoral CD90+CD73+ cells 5 days after cultured with SEB-activated CD3+ T cells in a transwell system. (g) Change in gMFI in CD127, CD107a and PD-1 expression in SEB-activated CFSE-labeled T cells after 5 days using a transwell system. (h) Detection of TNFα and IFNγ in the supernatants. Data in c, e, f, g, and h determined by flow cytometry and presented as mean ± SD. Significant differences in c, e, f, g and h calculated using one way ANOVA following by post hoc Tukey's range test. ns, not significant.

We next investigated whether peritumoral CD90+CD73+ cells negatively regulate T cell function. We performed *in vitro* functional assays using CFSE-labelled CD3+ T cells derived from blood of healthy donors stimulated with staphylococcus enterotoxin B (SEB) (Fig. 6a). We chose the microbial protein SEB to activate T cells based on its function as a superantigen and immune activator inducing strong T cell stimulation via binding directly to T cell receptor (TCR) and MHC-II receptor.^38^ Using co-culture conditions, peritumoral CD90+CD73+ cells from both LUSC and LUAD specimens suppressed SEB-induced proliferation of CFSE-labeled CD3+ T cells at a 1:1 and 5:1 ratio (T cells: CD90+CD73+ cells), irrespective of immune priming with TNFα/IFNγ (Fig. 6b,c). Supernatants (SN) from IP peritumoral CD90+CD73+ cells also effectively blocked the proliferation of SEB-activated T cells (Fig. 6c). The potent inhibition was confirmed using a transwell culture system (Fig. 6d,e). PD-L1 was upregulated in peritumoral CD90+CD73+ mesenchymal cells at a ratio of 1:1 (Fig. 6f), which was associated with a decrease in the expression of the activation markers PD-1 and CD107a, as well as the homeostatic regulator CD127 (Fig. 6g). These phenotypic changes coincided with suppression in the release of cytotoxic cytokines TNFα and IFNγ (Fig. 6h). Together, these data show that peritumoral CD90+CD73+ cells are naturally equipped with an immune regulatory function. Therefore, in a highly inflamed TME, peritumoral CD90+CD73+ mesenchymal cells may restrict host antitumor T cell responses in an adaptive manner resulting in local immunosuppression.

### Neutralization of PD-L1 and TFGβ1 combined with IDO1 inhibition in peritumoral CD90+CD73+ cells relieves T cell immune suppression

**Figure 7:**
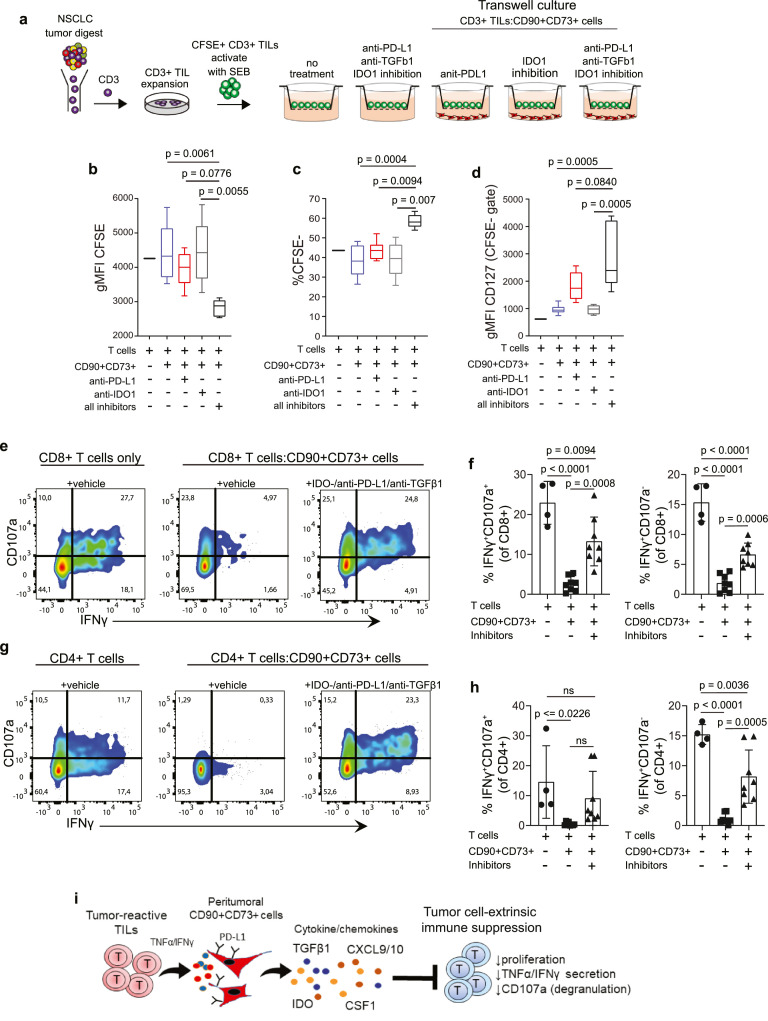
**Combined neutralization of PD-L1 and TGFβ1 with inhibition of IDO1 reverses immune suppression in peritumoral CD90+CD73+ cells**. (a) Schematic representation of experimental set-up to examine the immune suppressive function of peritumoral CD90+CD73+ cells on CD3+ tumour-infiltrating lymphocytes (TILs). (b–d) Box and whisker graphs showing change in gMFI CFSE (b), % CFSE- (c) and gMFI CD127 (IL7R) (d) in CD3+ TILs in the presence of peritumoral CD90+CD73+ cells after 5 days using a transwell system. Representative flow cytometric histogram showing CD107a and IFNγ expression in CD8+ (e) and CD4+ (g) TILs following activation with SEB (1 µg/mL) alone or in the presence of immune primed (IP) peritumoral CD90+CD73+ cells. (f,h) Bar graphs showing % of CD107a+IFNγ+ and CD107a-IFNγ+ CD8+ (f) and CD4+ (h) T cells. For co-culture conditions n = 8 in total. n = 4 for LUAD and n = 4 for LUSC. Data in b-d and f,h determined by flow cytometry. (i) Scheme of how peritumoral CD90+CD73+ cells within the TME target TILs for immune suppression. Data presented as mean ± SD. Significant differences in b, c, d, f and h calculated using one way ANOVA following by post hoc Tukey's range test. ns, not significant.

Next, we investigated the functional consequences of peritumoral CD90+CD73+ cells on tumour-infiltrating lymphocytes (TILs) isolated from patient tumour material (Fig. 7a). Peritumoral CD90+CD73+ cells abrogated the proliferation of SEB-activated CFSE-labeled CD3+ TILs (Fig. 7b,c). Cocultures treated with neutralizing antibodies against PD-L1 or chemical inhibition of IDO1 alone resulted in marginal changes in proliferation and no change in expression of CD127, a key homeostatic regulator of T cell survival and proliferation via IL-7 signalling.^39^ However, combined neutralization of PD-L1 and TGFβ1 with inhibition of IDO1 resulted in the restoration of TIL proliferation while increasing expression of CD127 (Fig. 7b–d). In contrast, combined neutralization of PD-L1 and TGFβ1 only, or neutralization of PD-L1 with IDO1 inhibition or neutralization of TGFβ1 with IDO1 inhibition only did not restore TIL proliferation (Fig. S7a,b). We also found that pretreatment of peritumoral CD90+CD73+ cells with α, β-Methyleneadenosine 5′-diphosphate (AMP-CP), an inhibitor of CD73 nucleoside activity, also failed to restore TIL function (Fig. S6a,b). In separate experiments, we isolated and expanded T cells from the uninvolved lung tissue. In cocultures of uninvolved lung-derived T cells activated with SEB with peritumoral CD90+CD73+ cells, we demonstrate that combined neutralization of PD-L1 and TGFβ1 with inhibition of IDO1 also restored degranulation (CD107a) and IFNγ secretion of CD8+ (Fig. 7e,f) and CD4+ T cells (Fig. 7g,h). In contrast, this was not observed after pretreating peritumoral CD90+CD73+ cells with combined blockade of PD-L1 with TGFβ1, PD-L1 with IDO1 inhibition or TGFβ1 with IDO1 inhibition or AMP-CP (Fig. S6c,d). Collectively, these data suggest that peritumoral CD90+CD73+ cells may serve a critical role in shaping T cell function in NSCLC. More importantly, our findings highlight the importance of blocking PD-L1 in combination with independent biological features of the mesenchymal compartment in the tumour stroma.

## Discussion

Since the prognostic and predictive role of PD-L1 staining in solid tumours remains uncertain,^40^ significant attention also has been placed on the abundance and localization of CD8+ T effector cells as a better prognostic and predictive biomarker due to their association with improved patient outcomes.^16^ In support of this, an IFNγ-driven T-cell inflamed gene expression profile is associated with improved response to PD-1 blockade with pembrolizumab across multiple tumour types.^22^ Using a core set of genes indicative of a CD8+ T cell effector phenotype, we observed a high enrichment of this IA signature score in NSCLC across the TCGA Pan-cancer cohort. These immunologically active tumours are enriched in a core set of genes defining an ECM/stromal signature. Importantly, a higher ECM/stromal to IA gene signature ratio is associated with poor survival. We further show that in primary resected NSCLC tumours, peritumoral cells that coexpress CD90 and CD73 are enriched in genes comprising the ECM/stromal gene signature, which were enhanced by TGFβ1. Peritumoral CD90+CD73+ cells found in both LUAD and LUSC samples can prevent the physiological activation of T cells using both cell contact and soluble mediators. Importantly, only combined PD-L1 and TGFβ1 blockade together with IDO1 inhibition was able to restore T cell function.

There is a growing body of evidence to support the TGFβ-responsive non-hematopoietic stromal compartment in regulating host antitumor immunity and response to ICB in solid tumours in humans.^17^, ^18^, ^19^^,^^41^ In human metastatic urothelial cancer, TGFβ-primed peritumoral fibroblasts function to exclude CD8+ TILs limiting the response to PD-L1 blockade using atezolizumab.^18^ The pan-fibroblast TGFβ response signature was highest in inflamed tumours or tumours where T cells were localized within the stroma. Using the EMT6 murine tumour model that recapitulates this immune-excluded tumour phenotype, combined blockade of PD-L1 and TGFβ reprogrammed peritumoral fibroblasts converted tumours from an excluded to inflamed phenotype resulting in a reduction in tumour burden.^18^ TGFβ-primed stroma has been implicated in the exclusion of TILs in a murine model of metastatic colon cancer.^41^ In support of this, a stroma-mediated source of immune resistance and lack of response to PD-1 blockade was reported in a separate cohort of patients with urothelial cancer.^19^ In both LUAD and LUSC tumour digests, not only are peritumoral CD90+CD73+ cells enriched in the ECM/stromal gene signature, which was amplified by TGFβ1, but these cells themselves are a major source of inflammatory-driven TGFβ1 secretion. Besides TGFβ1, inflammatory-driven CD90+CD73+ peritumoral cells secrete a wide range of cytokines belonging to the C-C and C-X-C chemokine family that are chemotactic for immune cells and also contribute to wound healing. The most abundant C-X-C chemokines secreted were CXCL9 and CXCL10, which are critical regulators of immune cell recruitment/activation via the CXCR3 axis in T cells, as well as NK cells.^42^ This paracrine axis could serve to attract and retain T cells in the stroma. As shown in fresh *ex vivo* slices of human lung tumours, the dense stroma acts as a physical barrier preventing T cells from infiltrating tumour islands, which was most prominent in the perivascular region.^14^ Moreover, CD90-positive mesenchymal stromal cells from human LUSC suppress NK cell cytotoxic function via the release of soluble prostaglandin E2 (PGE2).^43^ The comparability between the mesenchymal populations is unclear, as plastic adherence was used in the aforementioned study compared with the prospective isolation of a discrete subpopulation of tumor-associated mesenchymal cella using a FACS-based approach in our study. Nonetheless, CD90-positive mesenchymal cells in both the perivascular and stromal regions of human lung NSCLC correlate with poor patient prognosis.^43^^,^^44^

Surprisingly, our study reveals that TNFα is a more potent driver of the stromal immune regulatory secretome than IFNγ. We could only reverse the suppressive function of peritumoral CD90+CD73+ mesenchymal cells by a combined blockade of PD-L1, TGFβ1 and IDO1 but not by using them as single agents. In human breast cancer, isolation of a FAP+PDGFRβ+ CAF subset was shown to promote survival and differentiation of inhibitory Tregs via both a cell contact and paracrine mechanism involving CXCL12^45^ and was extended to mesenchymal high-grade serous ovarian cancers.^46^ Recently, a population of CD90+ mesenchymal cells found in human and mouse breast tumours contained subsets of FAP+PDPN+ and FAP+PDPN- cells with tumor growth-promoting properties; however, only the CD90+FAP+PDPN+ subset was immune suppressive.^47^ Recently, single-cell analysis of human breast tumours identified a cluster of FAP+ fibroblasts whereby subpopulations enriched in ECM proteins and TGFβ1 signalling were linked with regulation of host immune response via upregulation of PD-1 and CTLA4 expression on Tregs.^48^ Importantly, these subclusters were further shown to be enriched in patients with metastatic melanoma and NSCLC that failed to respond to ICB compared with responders.

Here, our findings highlight ways by which a highly inflamed TME may drive the transition of peritumoral CD90+CD73+ cells into critical regulators of T effector cell function and provide a potential means of targeting this axis to boost host antitumor immunity and response to ICB in the setting of NSCLC. However, our study has several limitations. First, we did not assess whether the relationship between IA and ECM/stromal signature scores on patient outcomes is associated with neoantigen burden and whether this differs based on histological subtype. In LUAD, tumours with a high clonal neoantigen burden are more homogenous and associated with improved response to ICB.^49^ Homogenous tumours with a high neoantigen burden are marked by an inflamed TME with significant upregulation of PD-L1 and IL-6.^50^ This was not observed in LUSC, which are more heterogeneous.^50^ Homogeneous LUAD tumours were also enriched in genes associated with a CD8+ T cell effector phenotype. Based on our analysis, the association between PD-L1 and the ECM/stromal gene signature was stronger in LUAD compared with LUSC. Moreover, IL-6 represented the most prominent cytokine secreted from both LUAD and LUSC-derived CD90+CD73+ peritumoral cells in response to TNFα. A comprehensive molecular analysis across several solid cancer subtypes supports a TGFβ-responsive stromal signature as an independent predictor of failure to ICB, irrespective of tumour mutational burden.^17^ Although our prelimary data show that simultaneous blockade of PD-L1, IDO and TGFβ1 restores, in part, TIL function, a deeper immunogenomic profiling is required to gain a greater understanding of the molecular underpinnings driving this response. Therefore, future studies require linking multifactorial cellular and molecular profiling together with neoantigen burden to identify subsets of patients that may benefit from immune checkpoint blockade combined with targeting independent biological features of the tumour stroma in NSCLC. Secondly, we were not able to address the contextual cues from cancer cells that drive the stromal directives. Human lung CD90+CD73+ cells support de novo vessel formation [25,26], resembling pericyte-like support cells.^51^ However, this capability becomes dyregualted in tumor-associated CD90+CD73+ cells.^27^ Here, TGFβ1 upregulates PDGFRβ expression in peritumoral CD90+CD73+ cells, which is invovled in the recruitment of pericytes to growing tumours via tumor-derived PDGF-BB.^21^ Therefore, whether PDGFRβ/PDGF-BB axis is invovled in the initial recruitment of resident CD90+CD73+ cells to growing tumors requires further investigation in a well-defined murine tumour model. Thirdly, while PD1/PD-L1 and CTLA4 are important immune checkpoints, the immunosuppressive adenosine molecule in the tumour stroma represents an emerging therapeutic target.^52^ Adenosine is derived from the breakdown of extracellular ATP coming from dying cancer cells and other sources as a result of hypoxia and celluar stress via two ectonucleotidases CD39 and CD73, which we show are co-expressed by peritumoral mesenchymal cells that also co-express PD-L1. We were not able to determine the contribution of these two ectonucleotidases to the immunosuppressive function of CD90+CD73+ peritumoral cells, as targeting CD73 alone was without any benefit. Despite this, the CD73/adenosine pathway is upregulated in both treatment naïve and resitant *EGFR*-mutant NSCLC, which is associated with features of immune suppression.^53^ While anti-CD73 therapy boosts the efficacy of anti-PD-L1 and anti-CTLA4 ICB in murine tumour models^54^, it is not clear whether additional targeting of CD39 would offer any additional benefit in NSCLC.^33^ Finally, the significance of blocking the enzymatic breakdown of intratumoral ATP causing rising intratumoural levels of eATP would depend heavily on intratumoral cell types present and their ability to uptake eATP together with expression levels of purinergic receptors.^55^ eATP can have direct immune stimulatory effects on several target immune cell populations including dendritic cells (DC) enhancing chemotaxis and maturation via eATP binding to the P2Y2 and P2X purinergic receptors.^55^ This would also offset the immunosuppressive function of eADO on DC maturation, thereby having a beneficial impact on the adaptive anti-tumor immune response. That said, the boost in eATP-dependent immunostimulation might be offset by a pro-tumorigenic effect of eATP directly on cancer cells. Intratumoral eATP binds to purinergic receptors such as P2X purinergic receptor 7 (P2RX7) located on malignant cells, which has been shown in melanoma.^56^ High intratumoral eATP would stimulate cancer cell metabolism in P2 × 7R positive cancer cells, which leads to a depletion of nutrients, acidification of the TME and recruitment of myeloid-derived suppressor cells that together would culminate in T cell anergy. Chen and colleagues^57^ demonstrate that cancer cells also internalize eATP via macropinocytosis, providing a much-needed energy source fueling cellular growth, metabolism and migration, which has been reported from both melanoma and NSCLC.^58^ Thus, removal of ATP from the extracellular space involving ATP consumption by highly proliferating lung cancer cells could potentially be more advantageous, as this would promote cancer cell metabolism via enhanced glucose flux satisfying the increase in energetic and biosynthetic demands.^59^ Moving forward, preclinical validation in intact organisms with a competent immune system will be required to determine whether there is a causal link between targeting features of peritumoral CD90+CD73+ cells and enhanced host antitumor immunity and response to ICB in NSCLC.^17^^,^^48^ Second, it may be necessary to test these combinatorial ICB together with strategies that target ATP/purinergic signaling with P2X antagonists on tumor growth and the potential for serious side effects.

## Author contributions

LW – assisted in study design, data collection, data analysis, data interpretation, created figures, edited manuscript

HY – assisted in data collection, data analysis, data interpretation, created figures and edited manuscript

PD – assisted in data collection, data analysis and edited manuscript

SB – assisted in data collection, data analysis, data interpretation and edited manuscript

FB – assisted with data collection and edited manuscript

CW – assisted with data collection and edited manuscript

TMM – assisted with data collection and edited manuscript

RP – assisted with data collection and edited manuscript

NH – assisted with data collection and edited manuscript

WS – assisted with data collection and edited manuscript

RAS – assisted with data collection, funding and edited manuscript

SRRH – study design, data collection, data analysis, data interpretation, created figures, wrote and edited manuscript

## Declaration of Competing Interest

The authors declare no potential conflicts of interest

## Supplementary materials

Image, application 1

Image, application 2

Image, application 3

Image, application 4

Image, application 5

Image, application 6

Image, application 7

Image, application 8
