# Supplementary figures and images for: Peritumoral CD90+CD73+ cells possess immunosuppressive features in human non-small cell lung cancer
Source: eBioMedicine. 2021 Nov 2;73:103664. doi: 10.1016/j.ebiom.2021.103664 (PMC8577354; doi:10.1016/j.ebiom.2021.103664)

Figure S1

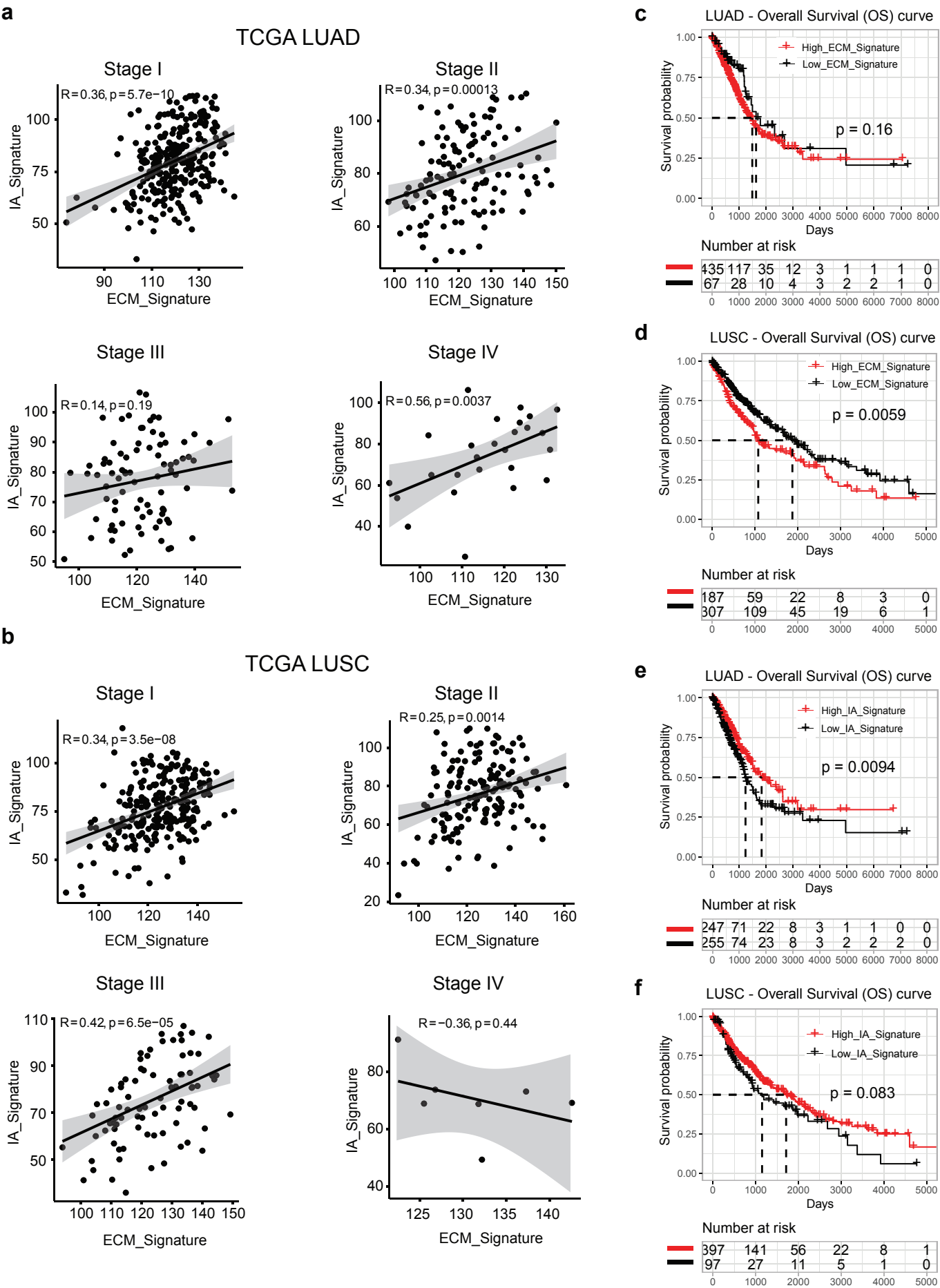

Supplement: Supplementary file 1 [file mmc1.pdf]

Figure S2

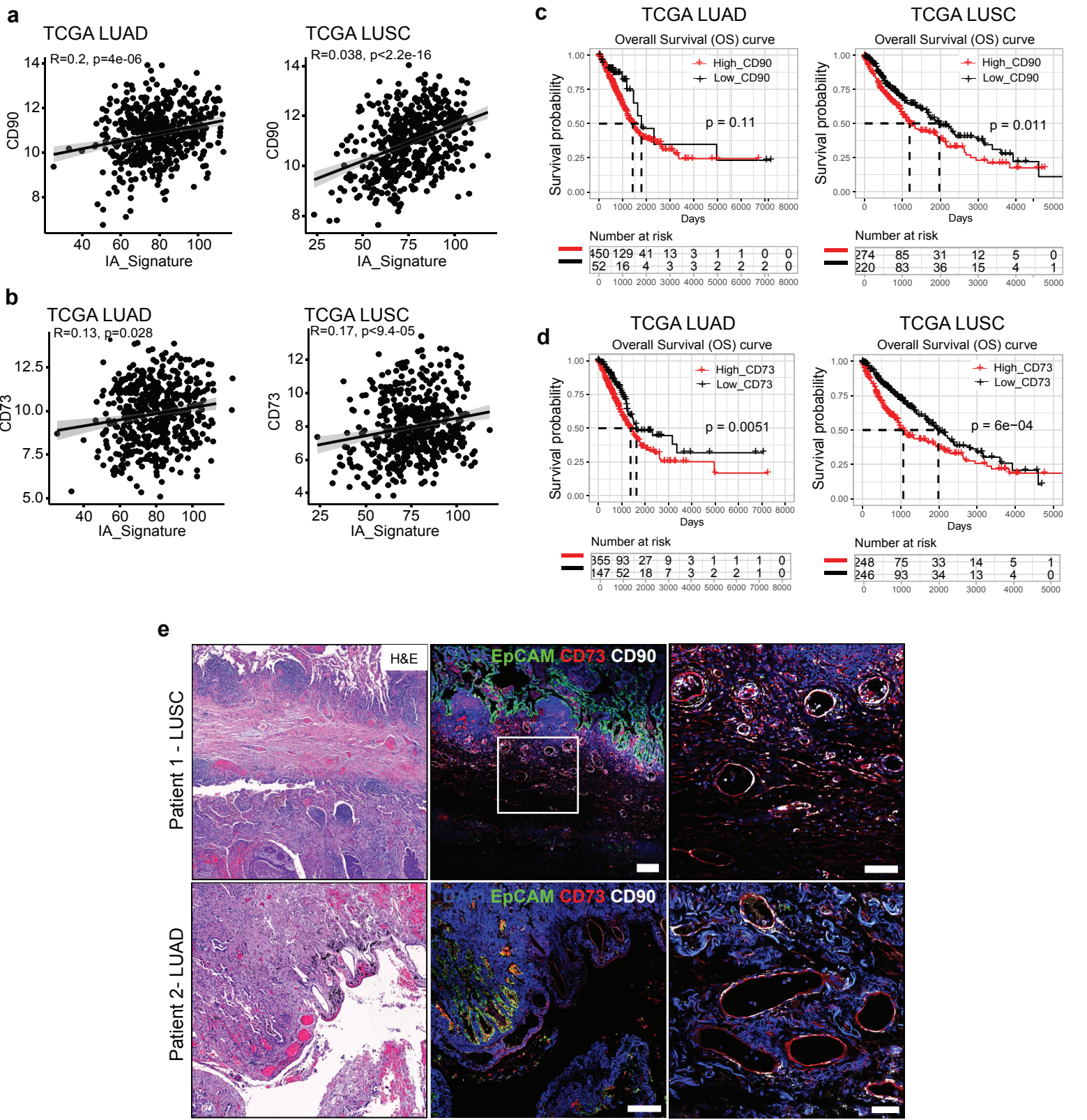

Supplement: Supplementary file 2 [file mmc2.pdf]

**Figure S3**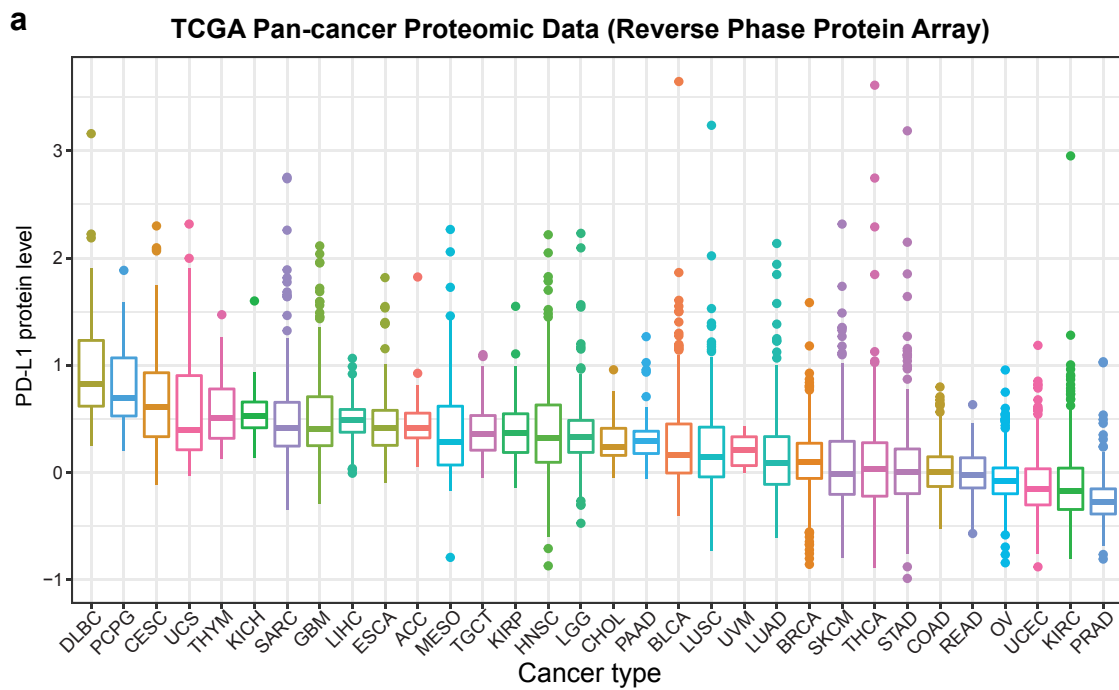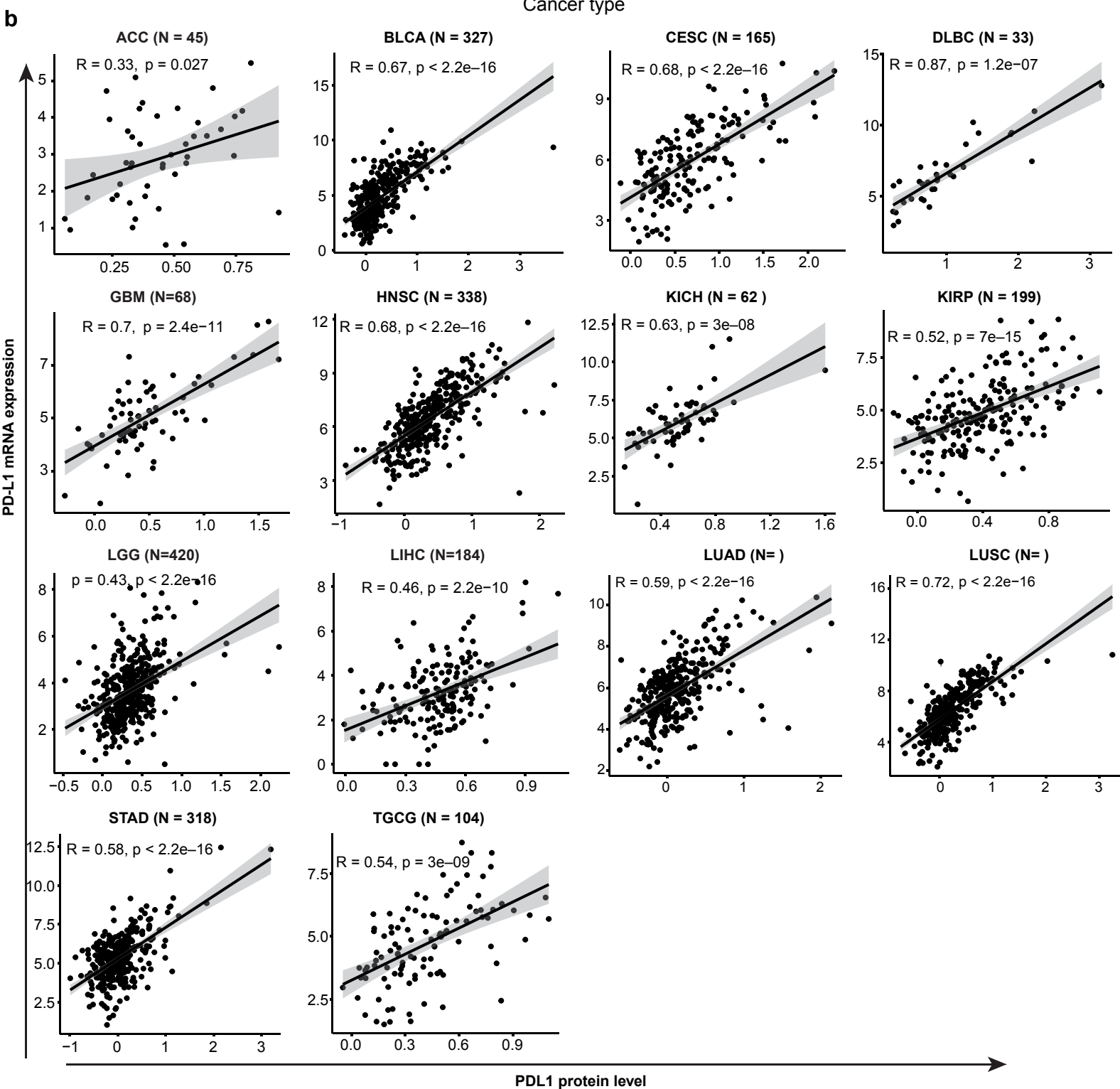

Supplement: Supplementary file 3 [file mmc3.pdf]

**Figure S4**

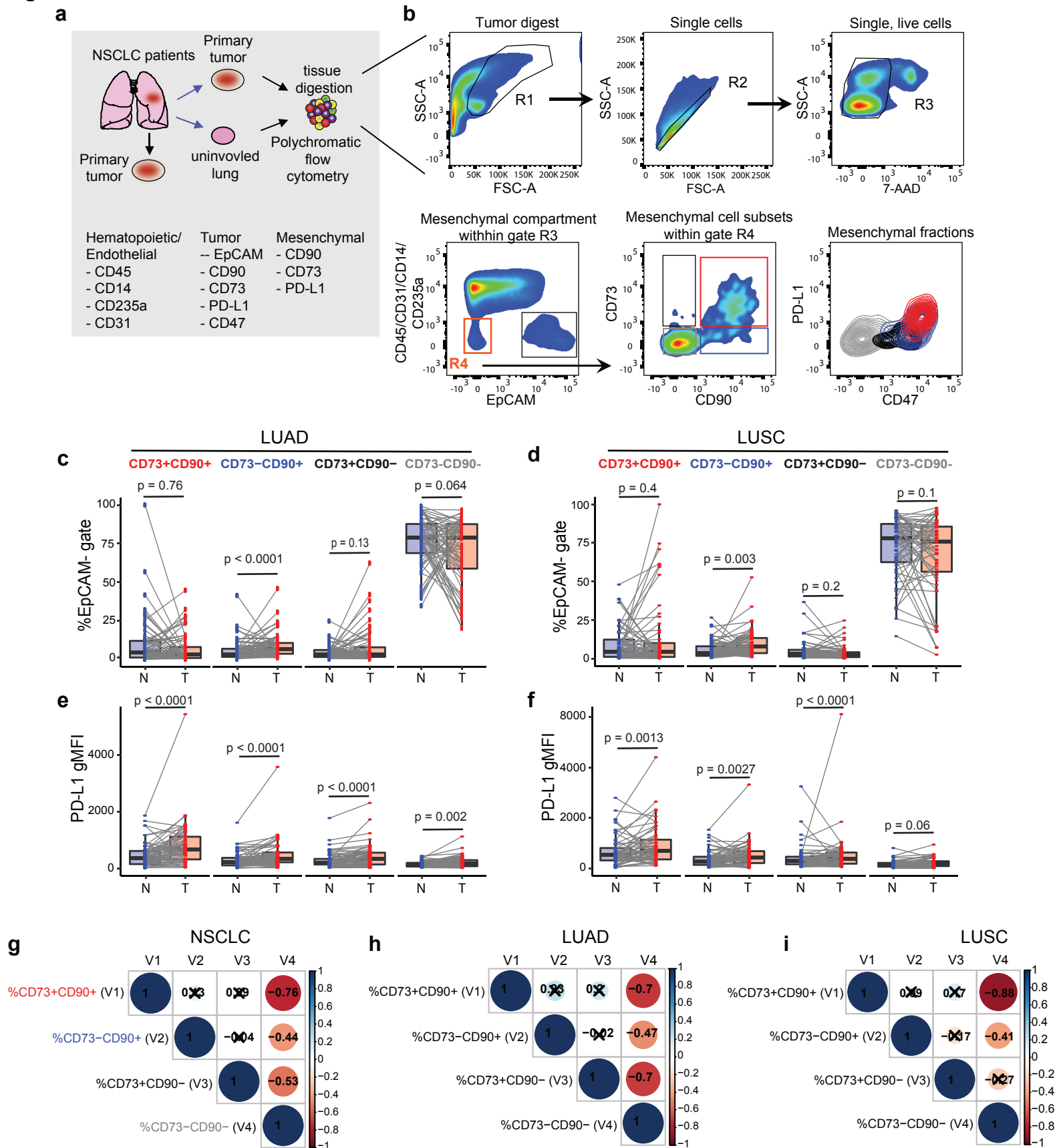

Supplement: Supplementary file 4 [file mmc4.pdf]

Figure S5

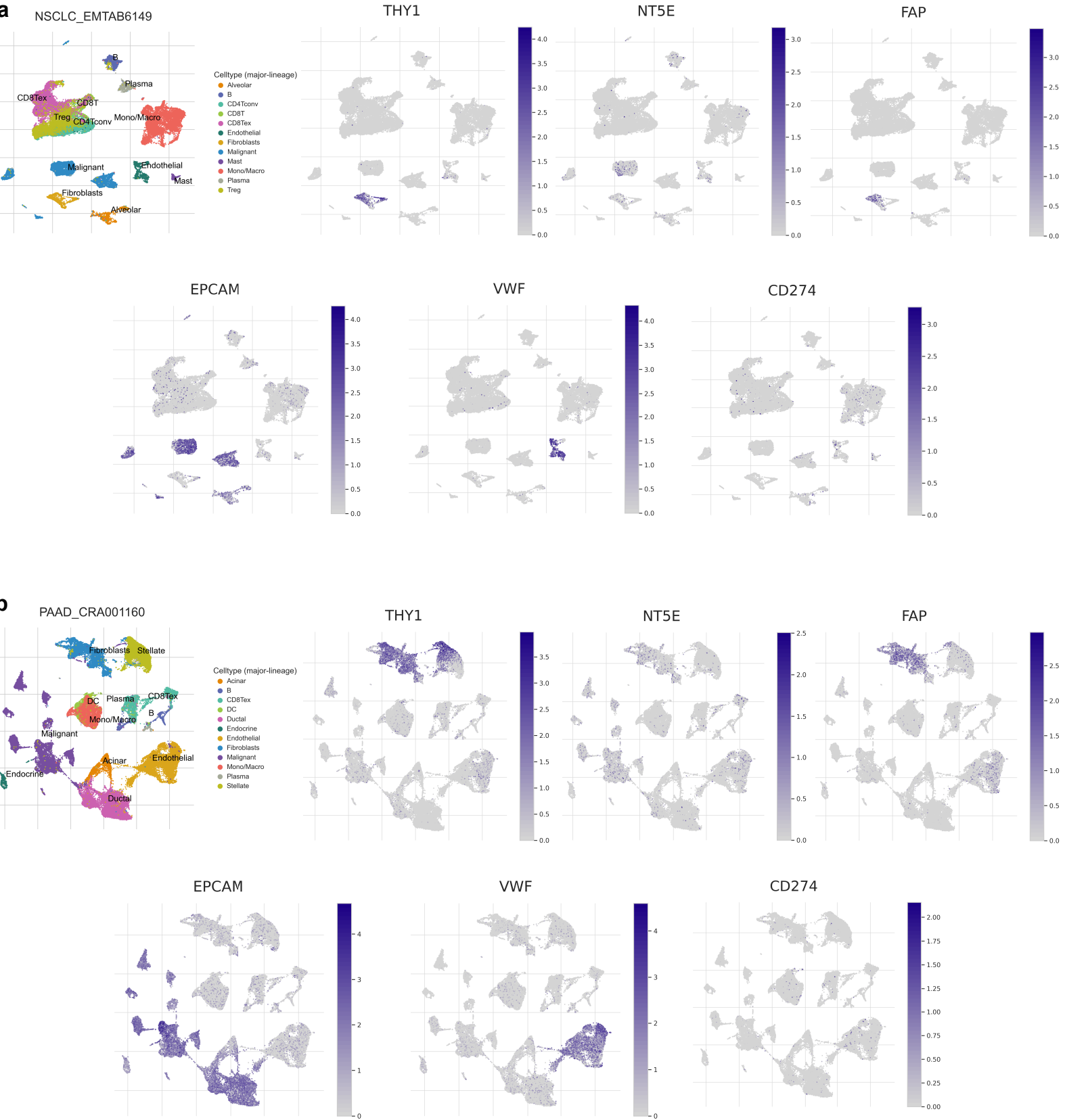

Supplement: Supplementary file 5 [file mmc5.pdf]

**Figure S6**

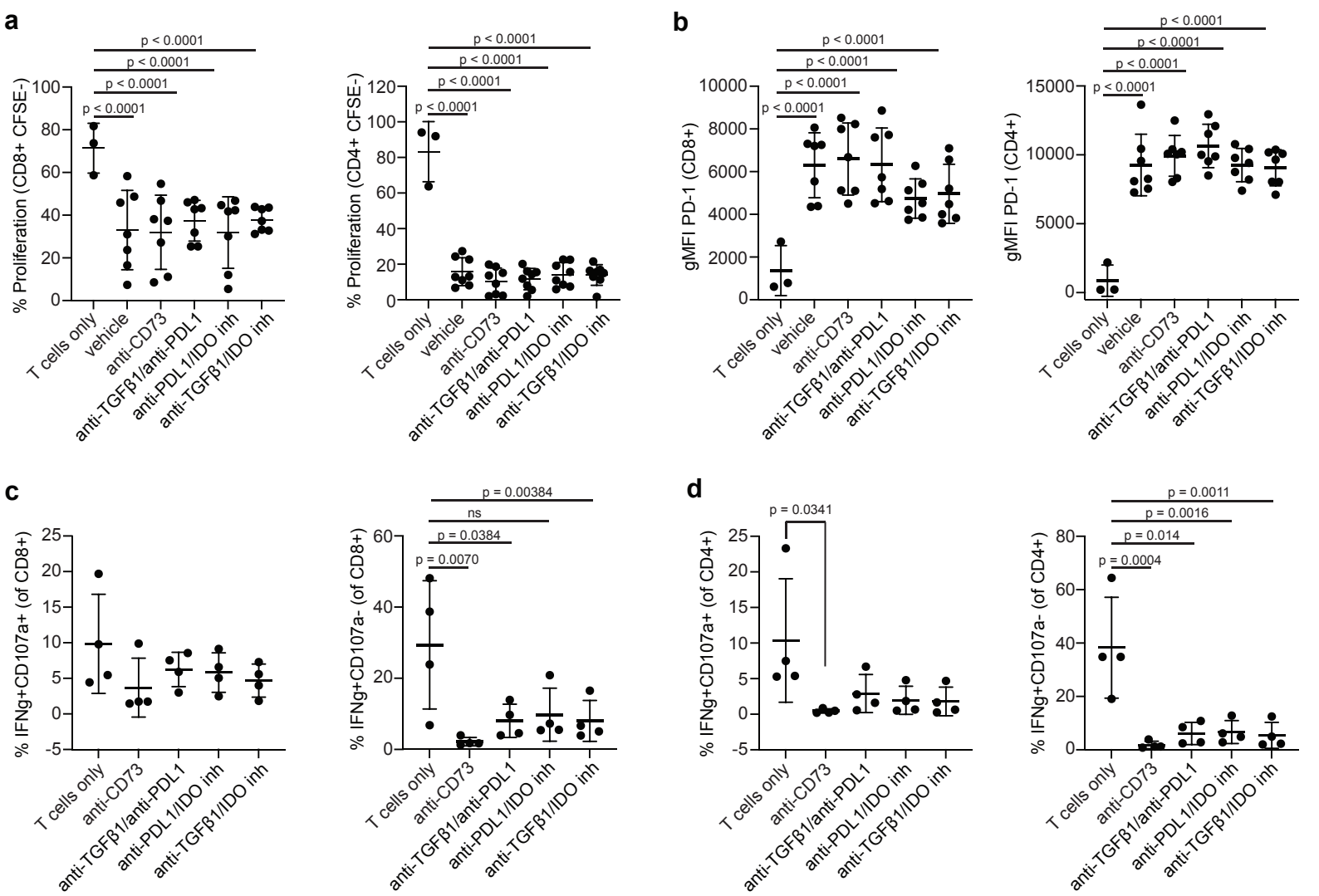

Supplement: Supplementary file 6 [file mmc6.pdf]

**Figure S7**

**a**

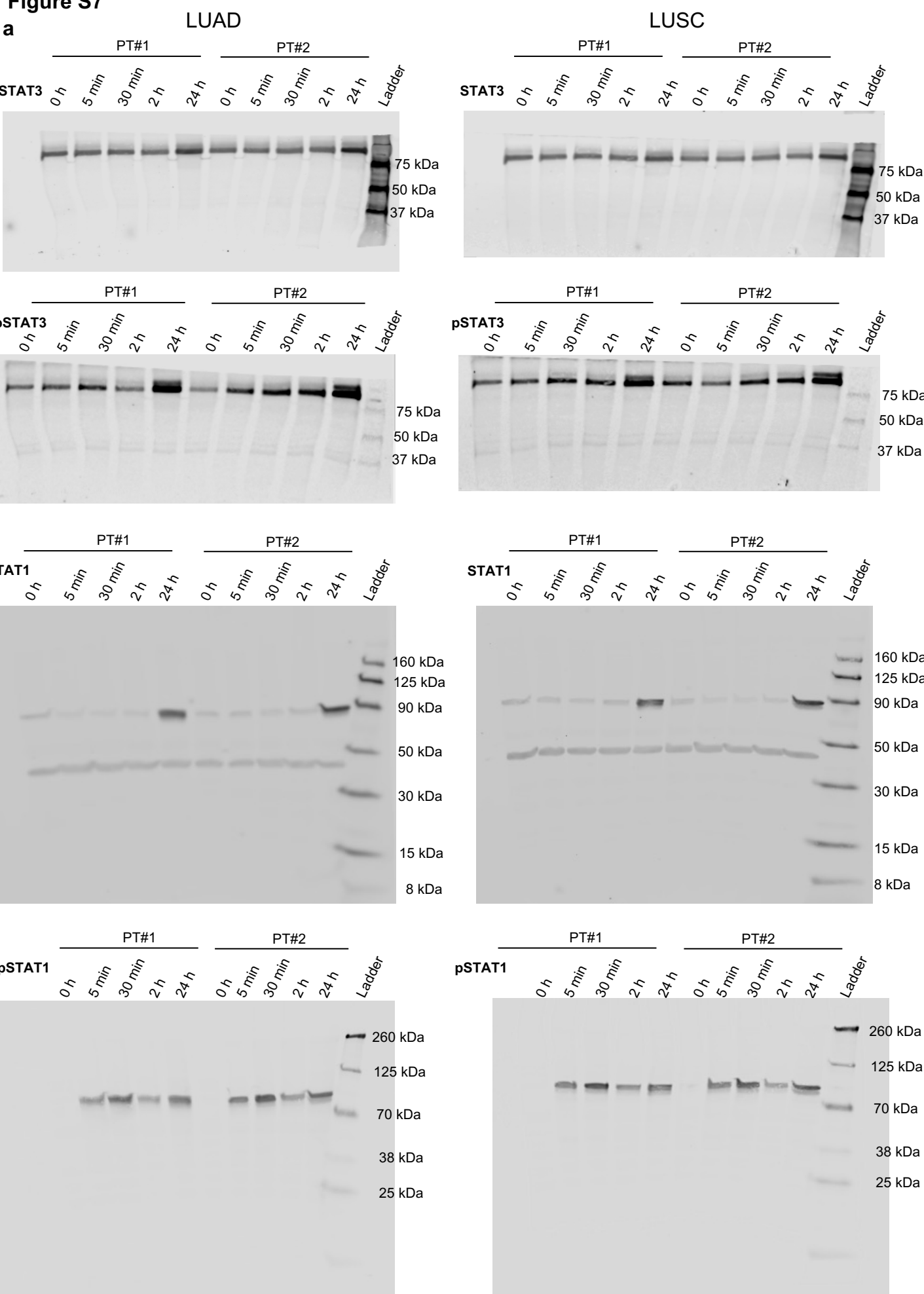

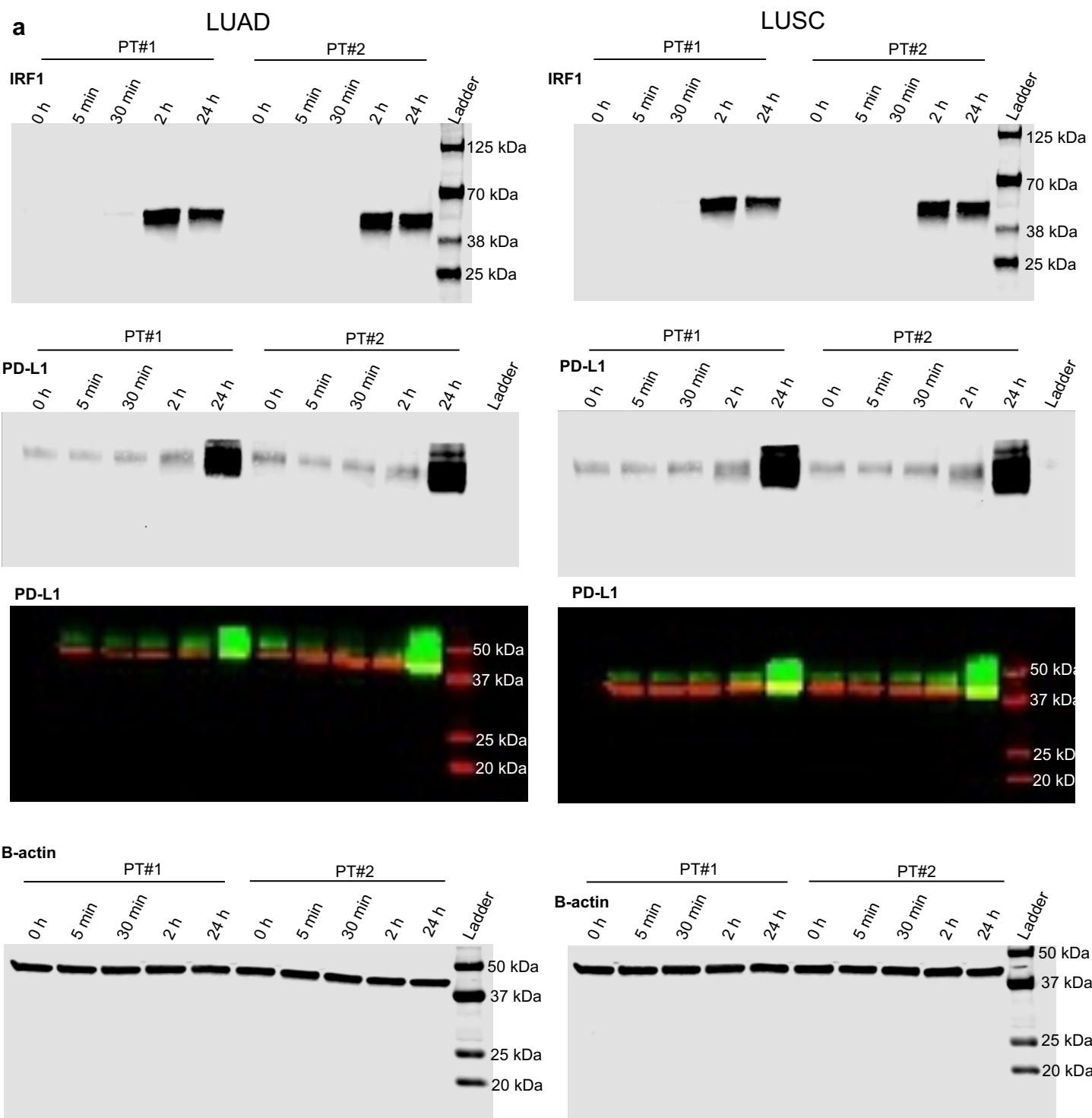

Supplement: Supplementary file 7 [file mmc7.pdf]
