# Supplementary material for: Peritumoral CD90+CD73+ cells possess immunosuppressive features in human non-small cell lung cancer
Source: eBioMedicine. 2021 Nov 2;73:103664. doi: 10.1016/j.ebiom.2021.103664 (PMC8577354; doi:10.1016/j.ebiom.2021.103664)
Supplement: Supplementary file 8 [file mmc8.docx]

**Methods**

**Study approval and acquisition of tissue samples**

The study was approved by Ethics Commission of the Canton of Bern (KEK-BE:042/2015). All patients gave informed written consent for use of surgical material for research purposes. Lung tumour samples were obtained from patients operated on for NSCLC with a curative intent at Bern University Hospital, Division of General Thoracic Surgery, September 2013 to January 2017. Unfixed surgical specimens were sent to the Institute of Pathology, University of Bern, where a pathologist dissected the tumor and matched non-tumorous lung tissue for further analysis. Cases included in the study were diagnosed as either LUAD (n = 64) or LUSC (n = 59). Twenty-nine patients received neoadjuvant treatment. Further clinic-pathological characteristics are provided in Table S1

**The Cancer Genome Atlas (TCGA) database and establishment of ECM and IIA gene signatures**

To generate an RNA-based metric to score ECM/stroma, we defined a gene set that contributes to ECM, EMT or stroma where there would be overlap in the expression found in the two main mesenchymal cell subsets in solid tumours: cancer-associated fibroblasts (CAFs) and pericytes. We chose hallmark CAF/pericyte genes VCAN, FAP, COL1A1, POSTN, THY1, FBLN1, and TGFβ1 mined three sources [1-3]. We also choose genes IL6, CSPG4, PDPN, HGF, SERPINE1 specific to lung perivascular-like cells that also would be involved in lung cancer [4, 5]. The defined gene set for the immune activation (IA) signature was chosen using hallmark genes with cytolytic activity expressed by CD8 TILs (GZMA, GZMB, GZMK, PRF1, IFNG, GNLY and IL2) [6, 7] and global activation of CD8 TILs based on single-cell RNA sequencing data in lung cancer patients [8], as well as our own internal dataset based on bulk RNA-sequencing of PD1+CD8+ TILs from NSCLC patients (Lipp J., et al. Research Square, 25 Jan 2021 DOI: 10.21203/rs.3.rs-152467/v1). Transcriptomic data were obtained from The Cancer Genome Atlas (TCGA) (https://portal.gdc.cancer.gov/projects/TCGA). After normalization (Limma package in the R) and log2 transformation, transcriptomic data were subjected to further analysis. Gene signature sore calculation: after scaling the genes expression value by Apply function in R, a sum of gene expression of the selected genes within the gene signature was then summarised as a single score for each sample. The gene expression and corresponding survival data were extracted for correlation and prognostic analysis using the corresponding packages in R (´corrplot´ and ´Hmisc´ packages for correlation analysis; 'maxstat', 'survival' and 'survminer' packages for prognostic analysis).

**Flow cytometric profiling and prospective cell isolation**

Generation of single-cell suspensions from tumour and matched non-tumorous lung tissue for flow cytometric profiling and prospective cell isolation using fluorescence-activated cell sorting (FACS) have been previously described [9]. Briefly, single cells were stained in buffer with Fc block (eBioscience) containing a panel of fluorescently conjugated human monoclonal antibodies directed at the following epitopes: CD45, CD14, CD31, CD235a, CD73, CD90, PD-L1, CD47 and EpCAM (see Table S2). To exclude cells from the analysis that stain for lineage markers not expressed by mesenchymal or epithelial cells, we created a dump gate pooling CD45, CD14, CD31, CD235a into one channel (see Figure S4 for full gating strategy). In a second smaller cohort, single cells were stained with a second antibody panel: CD45, CD14, CD235a, CD31, CD73, CD90, CD39, PD-L1, PDGFRα and EpCAM (see Table S2). To exclude cells from the analysis that stain for lineage markers not expressed by mesenchymal, endothelial or epithelial cells, we created a dump gate pooling CD45, CD14, CD235a into one channel. In this second smaller cohort, single cells were stained with a second antibody panel consisting of: Dump channel (CD45, CD14, CD235a), CD31, CD73, CD90, CD39, PD-L1, PDGFRα and EpCAM (see Table S2). Flow cytometric profiling of the mesenchymal compartment of single-cell digests of tumour and matched uninvolved lung tissue was performed using a BD FACS LSRII (BD Biosciences). For analysis, a minimum 5x10^5^ live events were collected and analyzed using FlowJo software ver10.7.1. PD-L1 and CD47 expression were measured as geometric mean fluorescence intensity (gMFI) in FlowJo. To prospectively isolate mesenchymal cells from the tumor and matched uninvolved tissue, single cells were stained as described above and sorted directly into collection buffer containing 20% FBS using a BD FACS Aria III or BD FACS Aria. Following this, cells were expanded in α-MEM (Sigma) supplemented with 1 % FBS (Invitrogen), 10 ng/ml of recombinant human bFGF (Gibco, Invitrogen), 20 ng/ml of recombinant EGF (Gibco, Invitrogen) and 1.25 mg of human insulin solution (Sigma) and 1X antimycotic/antibiotic (Gibco, Invitrogen). Culture expanded cells were used for all downstream experiments.

**Immunohistochemistry**

Serial sections from formalin-fixed and paraffin-embedded LUAD (n = 22) and LUSC (n = 24) cases were stained for PD-L1 and CD47. Immunohistochemical staining was performed using an automated immunostainer (Bond III, Leica Biosystems, Muttenz, Switzerland) using the following antibodies: anti-human PD-L1 and anti-human CD47 (clone B6H12, Santa Cruz, San Diego, USA) at a dilution of 1:20. Following this, sections were incubated with primary antibodies at room temperature for 15 minutes, followed by incubation with the secondary antibody using the Bond Polymer Refine Kit with 3-3’-Diaminobenzidine-DAB as chromogen (Leica Biosystems), counterstained with hematoxylin and mounted in Aquatex (Merck, Darmstadt, Germany). In a subset of patients, additional sections were also stained for anti-FAP (clone 1E5, Abcam) at 1:1500 dilution.

**Immunofluorescence staining on NSCLC sections**

5 µm sections were stained with a panel of human monoclonal antibodies targeting EpCAM, CD73 and CD90 (see Table 2). Following antigen retrieval using citrate buffer (10 mM, pH 5.5), samples were washed 3X in TBS buffer. Afterwards, slides were incubated for 30 min at room temperature in blocking solution (3% goat serum, 0.5% casein, 0.1% NaN_3_ in TBS buffer). Primary antibodies were added and slides were incubated overnight in a humidified chamber at 4°C. Afterwards, slides were washed 3X in TBS + 0.1% Tween 20. Secondary antibodies (See Table S3) diluted in TBS + 0.1% Tween 20 were applied in 100 µl aliquots to the slides and incubated for 3 hours in a humidified chamber at room temperature. Following staining, all slides were washed 2X in TBS + 0.1% Tween 20 and 100 µl of 4Ꞌ,6-diamidino-2-phenylindol (DAPI) (Molecular Probes, Invitrogen) solution was applied per slide for 30 seconds to counterstain nuclei. Slides were covered with EMS Shield Mount with anti-fading agent DABCO™ (Electron Microscopy Sciences). High-resolution images were acquired using a Zeiss LSM 710 Confocal Microscope using the Zen software program (Zeiss, Germany). Images were collected as lsm files and imported into Imaris software Ver 7.6 (Bitplane, CH).

**Immune priming CD90+CD73+ mesenchymal cells and and generation of conditioned media**

To generate conditioned media, 1x10^6^ CD90+CD73+ cells sorted from tumour digests were cultured in serum-free media for 24 hours prior to treatment with recombinant human TNFα (50 ng/ml, Gibco) together with recombinant human IFNγ (50 ng/ml, Gibco) or vehicle. After 24 hours cell supernatants were collected, centrifuged at 500 g to remove debris. Following this, cell-free supernatants were collected and placed in 3 KDa Amicon ultracentrifuge tubes (Merck, Darmstadt, Germany) and centrifuged at 3200 g for 25 min at 18°C. Supernatants were collected and protein concentration was determined using the Pierce BCA Protein Assay kit (ThermoFisher Scientific) according to the manufacturer’s instructions. Supernatants were aliquoted at known protein concentrations and frozen at -150°C until further use.

**Isolation and expansion of T cells**

Peripheral blood mononuclear cells (MNCs) were isolated from healthy blood bank donors using Ficoll-PaqueTM Plus (GE Healthcare Life Sciences, Switzerland) density gradient centrifugation. Following this, highly purified CD3+ T cells were isolated from the MNC pellet using the EasySep™ Human T Cell Isolation Kit (StemCell Technologies) according to the manufacturer’s instructions. Single cells were counted and cryofrozen and stored at -150°C until further use. To isolate T cells from resected NSCLC tumour digests, single tumour cell suspensions were stained with anti-human CD3 antibody and live/dead cell discrimination using 7-AAD (Invitrogen). CD3+ T cells were sorted using a BD FACS ARIA III (BD Biosciences) and were expanded using an expansion media (Immunocult, Stem cell Technologies) consisting of 10ng/mL of recombinant human IL-2 (StemCell Technologies), 10ng/mL of IL-7 (StemCell Technologies) and IL-15 (StemCell Technologies) with 2.5% human AB serum (Sigma) and anti-CD3/CD28/CD2 beads (StemCell Technologies). Cells were grown in low-adherent plates. Fresh media changes were made every six days until colonies of T cells were evident. Single cells were counted and cryofrozen and stored at -150°C until further use.

**Coculture and transwell T cell-activation assay**

Healthy donor CD3+ T cells were labelled with 0.5 µM CFSE (eBioscience) according to the manufacturer’s instructions. For coculture experiments, CFSE-labeled CD3+ T cells (2x10^5^) were stimulated with 1 µg/ml Staphylococcal Enterotoxin B (Sigma-Aldrich) in 96-wells U-bottom plates in T cell media (Immunocult XF, StemCell Technologies) supplemented with 10 ng/ml of recombinant human IL-2 (Peprotech) at 37°C and 5% CO_2_. Separately, tumour-derived CD90+CD73+ mesenchymal cells were gamma-irradiated (10 Gy) and in separate wells, 4x10^4^ (5:1) or 2x10^5^ (1:1) cells were added to wells containing 2x10^5^ CFSE-labeled T cells activated with SEB. Following this, co-cultures were treated with 50 ng/ml of rhTNFα (Gibco) and 50 ng/ml of rhIFNγ (Gibco) or vehicle. For transwell cultures, gamma-irradiated (10 Gy) tumour-associated CD90+CD73+ cells were prepared as described above. 2x10^5^ CFSE-labeled CD3+ T cells were seeded onto the top well and 2x10^5^ CD90+CD73+ cells in bottom well of 24-well culture inserts and incubated with 600 µl of T cell media (Immunocult). Cells were activated with SEB as described above. After 5 days of culture at 37°C and 5% CO2, cell-free supernatant was collected, frozen and stored at -20°C for later analysis. Cells were harvested and were stained with a cocktail of antibodies: anti-human CD45, anti-human CD4, anti-human CD8, anti-human CD127 and anti-human PD-1. Cytotoxic degranulation was detected by the addition of anti-human CD107a (eBioscience) [10] and cell viability using 7-AAD (eBioscience). Cells were analyzed for proliferation using CFSE-dilution patterns and for phenotype on a BD LSRII (BD Biosciences, San Jose, CA). For analysis of phenotypic markers, we used FlowJo software ver 10.7.1 (TreeStar) and precursor frequencies of activated T cells were calculated using ModFit LT® software (Verity Software House, USA). A minimum of 1x10^5^ events were collected for analysis. TNFα and IFNγ production was measured in the culture supernatants by ELISA according to the manufacturer’s instructions (Bio-Plex ProTM Human Chemokine assay, Bio-Rad, Austria). For blocking experiments, cells were treated with 50 µM of norharmane (Sigma), an IDO1 inhibitor, 10 µg of human PD-L1 neutralizing antibody (BPS Bioscience) and 2.5 µg of human TGFβ1 neutralizing antibody (MAB240, clone 9016, R&D Systems) for 3 hours alone or in various combinations. In separate wells, cells were treated with 10 µM of α, β-Methyleneadenosine 5′-diphosphate (AMP-CP, Sigma-Aldrich), an inhibitor of CD73 nucleoside activity. Afterwards, 2x10^5^ CFSE-labeled CD3+ cells were seeded per well and activated with SEB, as described above. At the end of 5 days, CFSE-labeled T cells were stained and analyzed as described above. To measure intracellular levels of IFNγ, cells were stained with near IR live/dead fixable dye (Invitrogen), according to the manufacturer’s instructions. Afterwards, cells were incubated with Fc block (Invitrogen) and in 100 µl of staining buffer with fluorochrome-conjugated CD107a-PE-TR. Afterwards, cells were washed and resuspended in 100 of Cytofix/Cytoperm™ solution for 20 min at 4°C. Cells were washed two times in 1X Perm/Wash™ solution. Afterwards, fixed/permeabilized cells were resuspended in 50 µl of Perm/Wash™ solution with anti-human IFNγ-APC antibody or appropriate isotype control and incubated at 4°C for 30 min in the dark. After staining, cells were washed 2X with 1X Perm/Wash™ solution and resuspended in PBS prior to flow cytometric analysis. A minimum of 50,000 events were collected using an LSR II BD Biosciences. FCS files were analyzed using FlowJo (ver 10.7.1).

**ELISA**

50x10^3^ peritumoral CD90+CD73+ cells (LUAD, n = 8; LUSC, n = 8, biological replicates) were cultured in serum-free media for 24 hours prior to treatment with vehicle (PBS), 50 ng/ml rhTNFα, 50 ng/ml rhIFNγ, or combined rhTNFα/rhIFNγ at 50 ng/ml each. After 24 hours, cell supernatants were collected, centrifuged at 500g to remove debris and frozen at -80°C. The levels of 40 cytokines/chemokines/growth factors were measured in cell-free supernatants using the Bio-Plex ProTM Human Chemokine assay (Bio-Rad) according to the manufacturer’s instructions.

**Western blotting**

Briefly, peritumoral CD90+CD73+ cells (LUAD, n = 2; LUSC, n = 2, biological replicates) were expanded in culture and 1 x 10^6^ were seeded in 10-cm cell culture dishes. Following 24 hours, cells were treated with rhTNFα (50 ng/ml) and rhIFNγ (50 ng/ml) for 5 minutes, 30 minutes, 2 hours and 24 hours in serum-free media. Protein was extracted using RIPA buffer (Thermo Fisher Scientific) containing a protease and phosphatase inhibitor cocktail (Thermo Fisher Scientific). Protein levels were determined using the BCA protein assay kit (Pierce). Proteins were transferred from SDS-PAGE gel to nitrocellulose membranes (Bio-Rad). Membranes were blocked with 10 ml of blocking buffer (LI-COR Biosciences) for 1 hour at room temperature. Following this, blots were incubated in diluted primary antibodies overnight at 4°C with gentle shaking in Odyssey blocking buffer (TBS) in the presence of 0.2% Tween 20. Blots were exposed to secondary antibodies in Odyssey blocking buffer (TBS) including 0.2% Tween 20 for 1 hour at room temperature with gentle shaking. Imaging of membranes was carried out using the Li-Cor Odyssey infrared imaging system. The following antibodies were used: anti-Stat1 (9H2) Mouse mAb (Cell Signaling Technology), anti-phospho-Stat1 (Tyr701) (58D6) Rabbit mAb (Cell Signaling Technology), anti-Stat3 (124H6) Mouse mAb (Cell Signaling Technology), anti-phospho-Stat3 (Tyr705) (D3A7) XP(R) Rabbit mAb (Cell Signaling Technology), anti-IRF-1 (D5E4) XP® Rabbit mAb (Cell Signaling Technology), anti-PD-L1 (E1L3N®) XP® Rabbit mAb (Cell Signaling Technology) and beta-actin (8H10D10) Mouse mAb (Cell Signaling Technology).

**Measurement of Annexin V and Propidium Iodide**

Early passage CD90+CD73+ cells and the NSCLC cell lines (LUAD: A549, H1299 and LUSC: H520, H1703) were plated at 10x10^4^ cells per well in 6 well plates in their respective media. After 24 hours, media was replaced with serum free media and cells were serum-starved for 24 hours. Serum starved cells were treated with vehicle or 2.5 µM cisplatin with 5 µM pemetrexed or 5 µM cisplatin with 10µM pemetrexed. Complete media changes with new drugs was added each day. After 72 hours, cells were stained with Annexin V/PI (Invitrogen) according to the manufacturer’s instructions. A minimum of 50,000 events were collected using a BD Bioscience LSR II (BD Biosciences). FCS files were analyzed using FlowJo (ver 10.7.1).

**Isolation of RNA and RT-qPCR**

Total RNA was extracted using RNeasy Mini Kit (Qiagen) to analyze gene expression using real time quantitative PCR (RT-qPCR). RT-qPCR was performed in triplicates with target-specific primers using TaqMan Gene Expression Assay (Applied Biosystems) on AB7500 FAST real-time PCR system (Applied Biosystems). Expression levels were normalized to 3 internal controls tested for expression stability across samples in each experiment using Expression Suite Software (Life Technologies). Relative expression was calculated by 2-ΔΔCT method. See supplemental Table S7 for the list of primers.

**Statistical analysis**

Data are expressed as mean ± SD. Comparisons between two groups were carried out using the parametric student’s two-tailed paired or unpaired t-test for normally distributed data. If data were not distributed normally, a nonparametric Wilcoxon signed-rank test was used between the two groups. One-way analysis of variance (ANOVA) followed by post hoc Tukey’ range test was used for analysis of more than two groups. The numbers of samples (biological replicates) per group (n), or the numbers of experiments (technical replicates) are specified in the figure legends. Data were analyzed using GraphPad Prism 8 software. For survival analysis, patients were grouped by gene expression, where ‘high’ and ‘low’ expression groups were stratified by the optimal cutoff value. Kapler-Meier analysis of a TCGA cohort of patients with LUSC and LUAD. Stratification of patients into high_extracellular matrix/stromal (ECM/stromal) (in red) and low_ECM/stromal (in black) or high_immune activation (IA) and low_IA is based on the optimal cutoff value of ECM/stromal gene signature score transcripts across all patients by using the surv_cutpoint function in R 'maxstat' package. Overall survival curves and cumulative hazard rates were analyzed and plotted by using R 'survival' and 'survminer' packages. The p-value is calculated using the log-rank test. All other statistical analyses were performed in the R Statistical Computing environment v3.3.1 (http://www.r-project.org). Statistical significance is accepted at p < 0.05.

**Figure S1. ECM/stromal genes and CD8 T cell activation genes are associated with tumorigenesis in NSCLC, as well as prognosi**s. (a,b). Plots showing the correlation between ECM/stromal gene signature with IA signature in TCGA LUAD (a) and LUSC (b) cohorts across all stages of lung cancer. (c-f) Unadjusted Kaplan-Meier survival curves from TCGA cohort of patients with LUAD and LUSC. Stratification of patients into (c,d) high_ECM (extracellular matrix) (in red) (c,d) and low_ECM (in black) or high_IA (in red) and low_IA (in black) (e,f) is based on the optimal cutoff value of ECM gene signature score and IA transcripts across all patients. Related to Figure 1.

**Figure S2. The TME in NSCLC marked by CD90 and CD73, as well as PD-L1**. Plots showing the correlation between CD90 (a) and CD73 (b) mRNA expression with IA_signature in NSCLC patients in TCGA. (c,d) Unadjusted Kaplan-Meier survival curves for NSCLC patients in TCGA divided into two groups by the CD90 (c) and CD73 (d) expression. (e) Confocal images showing the location of CD90 (white), CD73 (red) cells in relationship to EpCAM (green) positive tumour cells in NSCLC patient samples. Nuclei are pseudocoloured blue. Scale bar 50 µM. Related to Figure 2.

**Figure S3. PD-L1 expression in NSCLC and other solid tumours.** (a) Boxplots showing PD-L1 protein level in various cancer types in TCGA. (b) Plots showing the correlation between PD-L1 mRNA and protein level in various solid tumours in TCGA.

**Figure S4**. **Tumour-associated mesenchymal cell subsets in pulmonary LUAD and LUSC**. (a) Schematic showing characterization of mesenchymal cell subsets using multiparametric flow cytometry. (b) Representative tumour digest showing bivariate plots selected for single cells (gate R1 and R2) were further subgated for single, live cells (gate R3, 7-AAD negative). Following this, single, live cells (gate R3) were subgated onto bivariate plots to discriminate mesenchymal from hematopoietic, endothelial and epithelial cells. The mesenchymal fraction in gate R4 (Lineage-EpCAM) was further subgated onto bivariate plots for CD73 and CD90. Colour-coded mesenchymal fractions were subgated onto bivariate plots for PD-L1 and CD47. (c,d) Frequency of subsets of EpCAM^-^ cells subgated based on CD90 and CD73 in NSCLC specimens (T) compared to matched non-tumorous lung tissue (N) pairs in LUAD (n = 64) (c) and LUSC (n = 59) (d). (e,f) Scatter plots showing geometric mean fluorescence intensity (gMFI) for PD-L1 on gated mesenchymal cell subsets NSCLC specimens (T) compared to matched non-tumorous lung tissue (N) pairs in LUAD (n = 64) (e) and LUSC (n = 57) (f). All data determined by flow cytometry. (g-i) Correlation matrix showing a high correlation between CD90-CD73- and CD90+CD73+ in (g) NSCLC patient samples irrespective of histological subtype and separated based on LUAD (h) and LUSC (i) histology. Significant differences in c-f calculated between tumour (T) and matched non-tumorous lung tissue pairs (N) using Wilcoxon matched-pairs signed-rank test. Related to Figure 3.

**Figure S5. Single-cell analysis reveals heterogeneity in ECM/stromal/mesenchymal marker sin NSCLC and PAAD.** (a,b) UMAP visualization of colour-coded clustering of all cells (left panels) in NSCLC (a) and pancreatic adenocarcinoma (PAAD) (b). Expression of individual cell markers is shown in these clusters on the right. Data were generated from the Tumor Immune Single-cell Hub (TISCH) scRNA-seq database [11].

**Figure S6. Peritumoral CD90+CD73+ cells suppress tumour-infiltrating lymphocytes (TILs)**. (a) Scatter plots showing proliferation of SEB-activated (500 ng/ml) CFSE-labeled CD8+ (left) and CD4+ TILs (right) cocultured with vehicle or IP CD90+CD73+ cells, as well as their respective treatment conditions. For coculture conditions n = 7 in total. (b) Scatter plots showing the change in PD-1 geometric mean fluorescence intensity (gMFI) in SEB-activated CFSE-labeled T cells. (c,d) Scatter plots showing % of IFNγ+CD107a+ and IFNγ+CD107a- CD8+ (c) and CD4+ (d) TILs. Data in a-d determined by flow cytometry. Data presented as mean ± SD. Significant differences in a-d calculated using one way ANOVA following by post hoc Tukey’s range test. ns, not significant.

**Figure S7. Uncropped Western blots for Figure 5.**

**Table S1. Patient Clinical Characteristics**

| **Total # of patients, n** | 132 |
| --- | --- |
| **Gender, n (%)** |  |
| male | 85 (64%) |
| female | 47 (36%) |
| **Age at diagnosis, years, mean (range)** | 66 (39 – 96) |
| **Histology** |  |
| squamous cell carcinoma | 63 (48%) |
| adenocarcinoma | 69 (52%) |
| **Stage at diagnosis** |  |
| I | 51 (39%) |
| II | 39 (29.5%) |
| IIII | 36 (27%) |
| IV | 6 (4.5%) |
| **Smoking Status, n (%)** |  |
| never | 15 (11%) |
| former | 112 (85%) |
| current | 89 (67%) |
| unknown | 4 (3%) |
| pack years, average (range) | 38 (5-100) |
| **Co-morbidity, n** |  |
| COPD | 18 (14%) |
| **Prior treatment, n (%)** | 31 (23%) |
| chemotherapy | 28 (21.2%) |
| radiotherapy | 2 (2%) |
| chemo/radiotherapy | 1 (0.8%) |
| **Patient survival, n (%)** |  |
| survival data known | 118 (89%) |
| survival data unknown | 14 (11%) |
| dead | 29 (22%) |
| alive | 89 (67%) |
| **Tumor recurrence, n (%)** | 15 (11%) |
|  |  |

**Table S2. List of antibodies used for FACS/flow cytometric analysis**

| Primary Antibodies for Flow Cytometry | Manufacturer | Clone | Catalog # |
| --- | --- | --- | --- |
| CD326 (EpCAM)-PE-Cy7 | eBioscience | 1B7 | 25-9326-42 |
| CD73-APC | eBioscience | AD2 | 17-0739-42 |
| CD73-APC-Cy7 | BioLegend | AD2 | 344022 |
| CD90-BV605 | BioLegend | 5E10 | 328128 |
| CD90-PE-TR | BD Biosciences | 5E10 | 562385 |
| CD45-eFluor®450 | eBioscience | 2D1 | 48-9459-42 |
| CD45-APC-Cy7 | ThermoFisher Scientific | 2D1 | MA5-38731 |
| CD14-eFluor®450 | eBioscience | 61D3 | 48-0149-42 |
| CD31-eFluor®450 | eBioscience | WM-59 | 48-0319-42 |
| CD235a-eFluor®450 | eBioscience | HIR2 | 48-9987-42 |
| 7-AAD | eBioscience |  | 00-6990-50 |
| CD274 (PD-L1)-PE | BD Biosciences | MIH1 | 557924 |
| CD274 (PD-L1)-APC | BD Biosciences | MIH1 | 563741 |
| CD274 (PD-L1)-PE-Cy5 | ThermoFisher Scientific | MIH1 | 15-5983-42 |
| CD47-BV786 | BD Biosciences | B6H12 | 563758 |
| CD39-BV421 | BD Biosciences | TU66 | 563679 |
| CD31-BV605 | BD Biosciences | WM59 | 562855 |
| CD140a-BUV395 | BD Biosciences | αR1 | 742670 |
| CD140b-BV421 | BD Biosciences | 28D4 | 564124 |
| CD4-BV786 | BioLegend | SK3 | 344642 |
| CD4-PE-Cy7 | BD Biosciences | SK3 | 557852 |
| CD4-BUV395 | BD Biosciences | SK3 | 563550 |
| CD8-Alexa Fluor® 700 | BD Biosciences | RPA-T8 | 561026 |
| CD8-BUV496 | BD Biosciences | SK1 | 741199 |
| CD8-APC-Cy7 | BD Biosciences | SK1 | 557834 |
| PD-1(CD279)-PE-Cy5 | ThermoFisher Scientific | J105 | 15-2799-42 |
| PD-1(CD279)-BV605 | Biolegend | NAT105 | 367426 |
| CD107a-PE | BioLegend | H4A3 | 328608 |
| CD107a- PE/Dazzle™ 594 | BioLegend | H4A3 | 328646 |
| CD127-BV711 | BD Biosciences | HIL-7R-M21 | 563165 |
| IFN γ -APC | BD Biosciences | 4S.B3 | 551385 |
| CellTrace™ CFSE | ThermoFisher Scientific |  | C34554 |
| Fixable Viability Stain 510 | BD Biosciences |  | 564406 |
| LIVE/DEAD™ Fixable Near-IR | ThermoFisher Scientific |  | L34976 |
| Zombie Green™ Fixable Viability Kit | BioLegend |  | 423112 |

**Table S3**. **List of primary antibodies used for IHC/IF**

| **Primary Antibodies** | **Clonality** | **Species** | **clone** | **Dilution** | **Company** | **Catalog #** |
| --- | --- | --- | --- | --- | --- | --- |
| anti-human PD-L1 | Monoclonal | Rabbit | E1L3N | 1:400 | Cell Signaling | 13684 |
| anti-human FAP | Monoclonal | Mouse | 1E5 | 1:1500 | Abcam | Ab54651 |
| anti-human EpCAM/TROP-1 | Polyclonal | goat |  | 1:100 | R&D Systems | AF960 |
| anti-human CD90/Thy1 | Monoclonal | mouse | 7E1B11 | 1:100 | Novus Biologicals | NBP2-37330 |
| Anti-human CD73 | Polyclonal | rabbit |  | 1:100 | Novus Biologicals | NBP1-85740 |

**Table S4. List of secondary antibodies used for IF**

| **Secondary Antibodies** | **Dilution** | **Manufacturer** | **cat#** |
| --- | --- | --- | --- |
| Goat anti-rabbit Alexa Fluor®488 | 1:100 | Life Technologies | A11034 |
| Goat anti-rabbit Alexa Fluor®546 | 1:100 | Life Technologies | A11035 |
| Goat anti-mouse Alexa Fluor®546 | 1:100 | Life Technologies | A11003 |
| Goat anti-mouse Alexa Fluor®647 | 1:100 | Life Technologies | A21236 |
| Donkey anti-rabbit Alexa Fluor®488 | 1:100 | Life Technologies | A21206 |
| Donkey anti-goat Alexa Fluor®633 | 1:100 | Life Technologies | A21082 |

**Table S5**. **List of primary antibodies used for Western blotting**.

| **Primary**  **antibody** | **Company** | **Catalog #** | **Dilution** |
| --- | --- | --- | --- |
| Stat1 (9H2) Mouse mAb | Cell Signaling Technology | 9176S | 1:1000 |
| Phospho-Stat1 (Tyr701) (58D6) Rabbit mAb | Cell Signaling Technology | 9167S | 1:1000 |
| Stat3 (124H6) Mouse mAb | Cell Signaling Technology | #9139 | 1:1000 |
| Phospho-Stat3 (Tyr705) (D3A7) XP(R) Rabbit mAb | Cell Signaling Technology | 9145P | 1:1000 |
| IRF-1 (D5E4) XP® Rabbit mAb | Cell Signaling Technology | #8478 | 1:1000 |
| PD-L1 (E1L3N®) XP® Rabbit mAb | Cell Signaling Technology | #13684 | 1:1000 |
| beta-Actin (8H10D10) Mouse mAb | Cell Signaling Technology | 3700S | 1:5000 |
| IRDye® 800CW secondary antibody | Li-cor | 926-32213 | 1:15000 |
| IRDye® 680RD secondary antibody | Li-cor | 926-68072 | 1:10000 |

**References**

1. Chakravarthy A, Khan L, Bensler NP, Bose P, De Carvalho DD. TGF-beta-associated extracellular matrix genes link cancer-associated fibroblasts to immune evasion and immunotherapy failure. Nat Commun. 2018;9(1):4692.

2. Tirosh I, Izar B, Prakadan SM, Wadsworth MH, 2nd, Treacy D, Trombetta JJ, et al. Dissecting the multicellular ecosystem of metastatic melanoma by single-cell RNA-seq. Science. 2016;352(6282):189-96.

3. Wang L, Saci A, Szabo PM, Chasalow SD, Castillo-Martin M, Domingo-Domenech J, et al. EMT- and stroma-related gene expression and resistance to PD-1 blockade in urothelial cancer. Nat Commun. 2018;9(1):3503.

4. Bichsel CA, Hall SR, Schmid RA, Guenat OT, Geiser T. Primary Human Lung Pericytes Support and Stabilize In Vitro Perfusable Microvessels. Tissue Eng Part A. 2015;21(15-16):2166-76.

5. Wang L, Dorn P, Zeinali S, Froment L, Berezowska S, Kocher GJ, et al. CD90(+)CD146(+) identifies a pulmonary mesenchymal cell subtype with both immune modulatory and perivascular-like function in postnatal human lung. Am J Physiol Lung Cell Mol Physiol. 2020;318(4):L813-L30.

6. Ayers M, Lunceford J, Nebozhyn M, Murphy E, Loboda A, Kaufman DR, et al. IFN-gamma-related mRNA profile predicts clinical response to PD-1 blockade. J Clin Invest. 2017;127(8):2930-40.

7. Rooney MS, Shukla SA, Wu CJ, Getz G, Hacohen N. Molecular and genetic properties of tumors associated with local immune cytolytic activity. Cell. 2015;160(1-2):48-61.

8. Guo X, Zhang Y, Zheng L, Zheng C, Song J, Zhang Q, et al. Global characterization of T cells in non-small-cell lung cancer by single-cell sequencing. Nat Med. 2018;24(7):978-85.

9. Bichsel CA, Wang L, Froment L, Berezowska S, Muller S, Dorn P, et al. Increased PD-L1 expression and IL-6 secretion characterize human lung tumor-derived perivascular-like cells that promote vascular leakage in a perfusable microvasculature model. Sci Rep. 2017;7(1):10636.

10. Betts MR, Brenchley JM, Price DA, De Rosa SC, Douek DC, Roederer M, et al. Sensitive and viable identification of antigen-specific CD8+ T cells by a flow cytometric assay for degranulation. J Immunol Methods. 2003;281(1-2):65-78.

11. Sun D, Wang J, Han Y, Dong X, Ge J, Zheng R, et al. TISCH: a comprehensive web resource enabling interactive single-cell transcriptome visualization of tumor microenvironment. Nucleic Acids Res. 2021;49(D1):D1420-D30.
